# Investigation of deep learning approaches for automated damage diagnostics in fiber metal laminates using Detectron2 and SAM

**DOI:** 10.3389/frai.2025.1599345

**Published:** 2025-08-25

**Authors:** Sanjeev Kumar, Stefan Bosse, Chirag Shah

**Affiliations:** ^1^Department of Mechanical Engineering, University of Bremen, Bremen, Germany; ^2^Department of Computer Science, University of Koblenz, Koblenz, Germany; ^3^Chair of Materials Science and Materials Testing (LMW), Faculty IV: School of Science and Technology, Institute for Materials Engineering, University of Siegen, Siegen, Germany

**Keywords:** damage diagnostics, segmentation, fiber metal laminate, deep learning, explainable artificial intelligence, integrated gradients, mask R-CNN, segment anything model

## Abstract

The impact damage is one of the major causes of structural failures in Fiber Metal Laminate (FML) plates, which are widely used in the aerospace and automotive industries due to their superior mechanical properties. Accurate detection, segmentation, and characterization of these damages are crucial for improved safety and reduced maintenance costs. This study proposes an automated approach to detect, segment, reconstruct, and characterize the damages in FML plates using state-of-the-art deep learning models: the Segment Anything Model (SAM) and the Mask Region-based Convolutional Neural Network (Mask R-CNN) implemented by the Detectron2 framework. A domain-adapted supervised learning process was applied to the X-ray CT dataset of damaged FML plates impacted with energies of 5J, 7.5J, 10J, and 12.5J. Mask R-CNN significantly outperformed SAM across all key performance metrics while offering around 8 times faster training and 80 times faster inference. Mask R-CNN also proved to have superior explainability for end-users. The lack of absolute ground truth data severely limits the scope of an absolute quantitative comparison, therefore highlighting the need for further studies. This study not only contributes to the area of damage diagnostics in composite materials but also provides insights into the comparative performance and explainability of advanced deep learning models, paving the way for applications in industrial inspection and quality assurance.

## 1 Introduction

Fiber metal laminates (FMLs) have emerged as increasingly vital materials in the quest for high-performance, lightweight solutions across aerospace (Etri et al., 2022), marine (Ayyadurai et al., 2024), and automotive (Xiao et al., 2023) industries. These materials, driven primarily by the aerospace industry over recent decades, consist of alternating layers of hybrid composite materials and metal alloys. The synergy between metal and polymer composites in FMLs results in enhanced mechanical properties compared to their individual components, thus offering high specific strength and high specific stiffness-to-weight ratio, superior fatigue resistance, load-bearing capacity, impact resistance, and exceptional corrosion resistance (Botelho et al., 2006). Notable examples of commercially available FMLs include ARALL (Aramid Reinforced Aluminum Laminate), GLARE (Glass Reinforced Aluminum Laminate), and CARALL (Carbon Reinforced Aluminum Laminate) (Sinmazçelik et al., 2011).

An impact damage is a significant concern for the structural integrity of aircraft. Throughout the lifecycle of an aircraft, it is exposed to dynamic impacts from various sources such as stones, debris, hailstorms, and collisions with ground equipment. These impacts may also occur during the production, assembly, and in-service maintenance processes. A study on 71, Boeing 747 aircrafts revealed that 13% of structural failures were due to off-plane impact loading from external objects (Starikov, 2013). The anisotropic nature of composite materials and the plasticity of metals in FMLs contribute to complex damage modes upon impact, potentially leading to catastrophic failures. In order to reduce the frequency and extent of such failures, the researchers have traditionally employed a range of techniques, both destructive and non-destructive, for the analysis of the impact damage. Destructive methods, such as optical and electron microscopy, provide detailed insights but also introduce new damage or relax residual stresses. Non-destructive evaluation (NDE) techniques, on the other hand, are a more preservation-oriented approach. It includes techniques such as 2D and 3D X-ray Computed Tomography (CT), ultrasonic C-scan, and eddy current mapping. Among these, X-ray CT stands out as a particularly promising in-field technique for damage analysis as it offers superior full-field resolution and three-dimensional representation (L'eonard et al., 2014; Sinmazçelik et al., 2011; Starikov, 2013). X-ray computed tomography (CT) images of FML material provide high-resolution 3-D imaging of the damage inside the FML material, but they also include artifacts, high-frequency noise, beam hardening, defective detector pixels, and scattering of X-rays. This makes the segmentation of damages hard with the traditionally used thresholding algorithms. These algorithms are manual and semi-manual in nature and often suffer from the operator's bias (Iassonov et al., 2009). L'eonard et al. (2017) utilized X-ray CT data to analyze key failure modes in fiber metal laminates, including aluminum necking, matrix cracking, and interlaminar delamination. Their approach employed semi-automated classical image processing techniques such as watershed algorithms, threshold segmentation, and distance transforms. However, these methods often fall short of capturing the complex features of impact damages, especially in regions with varying contrasts between metals and composite matrices. Additionally, these techniques are susceptible to noise and require manual parameter tuning, making them time-consuming and potentially error-prone.

An important aspect of the damage diagnostics in materials is to distinguish between defects and damage. A defect can be considered just as a material deviation from a baseline model, but with unknown or unpredictable correlation with material and structural failure. e.g., pores in die-casted metal components are always present and considered as a material feature and not a damage. Damages can be considered either as a pre-condition or a final outcome of a material failure. For our work, the classification of material deviations in defects and damages is not relevant. A comprehensive damage classification has been presented in a former work dealing with the classification of defects and damages in hybrid and composite materials (Shah et al., 2022). Furthermore, in the context of this work, impact damage has been investigated to facilitate the broader scope of the ongoing research work toward the detection and identification of such damage for damage diagnostics. Classification of these impact damages remains challenging due to their strong dependence on the material layups and configurations. The damage mechanisms resulting due to an impact damage could be present in all the specimens and a classification scheme based solely on the presence or absence of specific damage features would not suffice the purpose of damage diagnostics where the severity of the damage is of critical importance rather than the presence or absence of some damage features. To overcome this, parallels were drawn from clinical medicine, where illness is often categorized by symptom severity, for e.g., fever may be classified as mild, moderate, or severe despite overlapping indicators across categories. Similarly, here in material science, for the classification of impact damages, these can be effectively classified into low, medium, and high intensity damages based on the severity (Shah et al., 2022). However, outside the scope of the impact damages, the material damages and defects can be characterized and modeled on different scales:

1. Micro-scale level, e.g., micro cracks;
2. Macro-scale level, e.g., delaminations in multi-layer materials;
3. Meso-scale level, i.e., damages or defects between micro- and macro-scale levels.

Therefore, structurally heterogeneous materials can be characterized based on multi-scale consideration, at the macro-, meso-, and micro-levels, as discussed fundamentally in Stukhlyak et al. (2015) for epoxy-composite materials. In this work, we consider automated characterization of damages on the macro-scale level. The cause of material failure (e.g., breakage) can be related to any scale, but Stukhlyak et al. (2015) discusses micro-scale defects as the root of material failures, too. The detection of macro-scale damages was chosen for multiple reasons:

1. Macro scale damage governs the maximum extent of the damage and is used as a sign for reliable damage detection. These can facilitate the damage detection, identification, and localization, which are extremely important for comprehensive damage diagnostics. Since these damaged features have a considerably larger extent and have a maximum influence on the signals during the damage diagnostics, they provide a confident signal change that could be reliable for the damage differentiation. Such macroscopic damage features include delamination, deformation in the metal layers, and local strain accumulation near the impact zone.
2. These macroscopic features can provide a realistic damage extent, thus facilitating a considerably easier determination of the damage severity. This damage severity, based on the extent of the macroscopic features such as delamination, can then be correlated with the Guided Ultrasonic Waves (GUW) signals for a reliable estimation of the damage. This forms the basis for the state of the damage studied in this study.
3. The geometric characterization of delamination volumes should be used to investigate damage diagnostics with Guided Ultrasonic Wave (GUW) measurements in the mid-frequency range (about 50–100 kHz base frequency), which are sensitive to larger material gaps (i.e., holes), but insensitive to spurious micro-scale defects like cracks due to the low wavelength in the centimeter range. Additionally, GUW signals are less sensitive to kissing bond delaminations due to the guidance of waves along and inside a layer. The GUW investigation is not considered in this paper, but it is considered as a data sink for the output data of this work (Maack et al., 2023).
4. Macro-scale defects in CT images can be easily and accurately labeled by experts and can be clearly distinguished from CT reconstruction artifacts, in contrast to micro-scale defects like cracks, which can be CT artifacts. The characterization of micro-scale defects is a challenge.
5. Geometric macro-scale areas and volumes are already local or global aggregate variables that can be used for damage assessment. Spurious micro-scale defects like cracks are single events with a local context that are hard to interpret.

Recent advancements in deep learning have shown promise in overcoming these limitations. Bordekar et al. (2023) applied machine learning algorithms to characterize pores using X-ray CT slices, although their approach may be limited when dealing with more complex damage features. Additionally, their approach to characterizing defects in material tends to overestimate volume and projected area measurements, as it utilizes convex hull fitting rather than the more precise concave hull fitting algorithm used in this paper. Several researchers have successfully employed deep learning for damage segmentation in X-ray CT data of the materials such as fiber-reinforced polymers (Helwing et al., 2022), concrete (Li et al., 2023; Lorenzoni et al., 2020; Tian et al., 2021; Dong and Qiao, 2021) etc. Li et al. (2023) utilized commercially available software for *in situ* deep learning model training, albeit with limited hyperparameter tuning capabilities. Other studies have explored various deep learning architectures such as Mask R-CNN (Tian et al., 2021), demonstrating the versatility of these approaches. Helwing et al. (2022) use a CNN model based on LeNet-5 to segment the damages such as matrix crack, interfiber failure, delaminations, etc. in fiber-reinforced polymers and prove it to be superior as compared to global thresholding. But their investigation was not focused on analyzing the damage morphology, calculation of its size. Kopp et al. (2022) applied deep learning models to segment micro-damages in heterogeneous fiber composite materials, focusing on comparing manual and automatic segmentation performance. Their approach combined U-Net and VGG16 architectures but did not leverage transfer learning techniques, which could potentially enhance complex feature learning efficiency. Wang et al. (2024) integrated deep learning with digital volume correlation (DVC) to characterize damage in Fiber Reinforced Plastic (FRP) composites, although their training process was notably time-intensive.

This study aims to develop an automated and accurate process for detecting, segmenting, and characterizing impact damages in FML materials using advanced deep learning algorithms applied to X-ray Computed Tomography (CT) image data. The importance of this approach lies in its potential to enhance the understanding of nature and the extent of impact damage, which can lead to the development of more resilient materials. Moreover, it could significantly improve the structural health monitoring processes, resulting in reduced maintenance times and costs without compromising quality. By leveraging cutting-edge AI technology, the research aims to make a significant contribution toward safer and more efficient aircraft operations. State-of-the-art deep learning algorithms show great potential for damage segmentation and analysis. Pretrained large Deep Learning (DL) models have been successfully adapted to detect damages in various domains such as roads, civil infrastructures, and rope structures (Rani et al., 2024; Pham et al., 2020; Ahmadi et al., 2023). Pretrained DL models offer faster feature extraction and adaptability with reduced training time and data requirements compared to training domain-specific models from scratch. In 2023, Meta AI Research introduced the Segment Anything Model (SAM), a foundational model for instance segmentation trained on a vast dataset of over one billion masks across 11 million images. SAM's suitability for versatile segmentation tasks, flexible prompting, and innovative data collection approach make it a robust tool for various applications (Kirillov et al., 2023; Ahmadi et al., 2023). A study by Gruber et al. (2024) demonstrates SAM's effectiveness in segmenting 3D CT data and performing material characterization. This paper aims to compare the suitability and performance of SAM with another deep learning framework the Detectron2, which uses the Mask R-CNN architecture. Detectron2, developed by Facebook AI Research, is an open-source object detection platform implemented in PyTorch. It supports a wide range of tasks, including object detection, instance segmentation, semantic segmentation, panoptic segmentation, and human pose prediction. Detectron2's modular design, coupled with pretrained models on massive datasets like COCO, Cityscapes, Pascal VOC, and ImageNet, facilitates powerful transfer learning for custom datasets.

This research paper presents a novel approach to analyze the impact damage in Fiber Metal Laminate (FML) materials by employing two state-of-the-art deep learning models: Segment Anything Model (SAM) and Mask R-CNN. Please note that the terms Detectron2 and Mask R-CNN are used interchangeably throughout the paper. Mask R-CNN is a deep learning model implemented using the Detectron2 framework. The study develops the process to automatically detect and segment impact damages within FML materials using these advanced algorithms, followed by a comprehensive comparative analysis of their performance and explainability. The paper characterizes damages through the application of the DBSCAN (Density-Based Spatial Clustering of Applications with Noise) clustering technique, combined with a concave hull fitting algorithm. This innovative combination of methods allows for a more detailed and accurate characterization of the detected damages. By integrating these cutting-edge techniques, the research aims to provide a more robust, efficient, and accurate approach to damage analysis in FML materials, potentially advancing the field of structural health monitoring and material science in aerospace applications.

## 2 Data-driven damage diagnostics

The specimens under investigation are FML plates with an alternating layer order of metal (aluminum) and fiber-resin (glass-epoxy) materials. The aluminum layer has a thickness of 0.2 mm, and the fiber-epoxy-resin layer of about 0.3 mm. The plates were cured under a vacuum. The impact damage was induced on the FML plate at the German Aerospace Center (DLR) in Braunschweig, and the CT data were collected at the MAPEX Center for Materials and Processes at the University of Bremen. The impact tests were conducted at varying energy levels of 5J, 7.5J, 10J, and 12.5J by shooting a projectile from the impact gun on the surface of the GLARE 5–5/4 with 54% Metal Volume Fraction (MVF) with the specimen thickness of 4mm and size 150 × 500 mm. The impactor was hemispherical with a diameter of 16 mm. The plates were reduced to a size of 50 × 50 mm around the impact location via water jet cutting. It contains the entire part of the damage. The smaller specimens were then investigated with X-ray computed tomography (CT) to capture the cross-sectional slices of the damage. The CT scans offer detailed visualization of the internal structure and damage patterns. The machine used for the scans was ZEISS Xradia Versa 520, having a total acquisition time for each specimen of 3.5 hours. The machine operated at a voltage of 110 kV and a power of 10 W, with an electron beam current of 91.3 A. The detector was positioned at a distance of 183 mm from the specimen, while the X-ray source was located 67 mm away. A total of 2,001 projections were acquired over a full 360-degree rotation, ensuring high-resolution imaging of the internal structure and damage patterns in the reduced specimens.

### 2.1 X-ray micro-CT

To assess different image feature marking and clustering algorithms and models, the root of the input data must be considered in more detail. The three-dimensional image slice stack is reconstructed from a set of *m* radial projections with an image size of *r* × *s* pixels. The pixel intensity is inversely proportional to the material density and X-ray absorption, i.e., air or vacuum is related to the highest, metal to the lowest intensity. The absorption (beam attenuation) depends on the material thickness along the projection beam, the material density, and the X-ray energy. The detector pixel intensity also depends on the X-ray energy (non-linearly). X-ray beams are commonly created by accelerated electrons creating Bremsstrahlung (electron-hull interaction), which is a continuous energy distribution up to the maximal tube acceleration voltage. The X-ray spectrum is superseded by narrow discrete lines originating from the nucleus of the anode target atoms. Due to the energy dependency of absorption and X-ray-light conversion materials, the original X-ray images show a blurring around material density variations (edges are washed out). The radial projection images are the input for the slice stack reconstruction, basically using the Radon transformation. This geometric transformation is highly sensitive to noise and reduced blurring in the input images. There are additional effects, e.g., dead pixels can create artifacts in the reconstructed images.

The resolution limit of the projection image is determined by:

1. The focal spot size diameter (FSD) of the X-ray tube;
2. The pixel size of the detector;
3. The energy distributions of the X-ray beam;
4. The geometric magnification given by the ratio of object-source and detector-source distances (and additional optical magnification);
5. Photon and electronics noise (limited Signal-to-noise ratio).

The quality and resolution of the reconstructed images (of size *m* × *r*) depends on:

1. All above limitations of the measuring system;
2. The number of projections (a higher number of projections reduces the delta angle between projection images);
3. Any image preprocessing;
4. Spatial frequency filtering before the Radon transformation is applied;
5. Any post-filtering of the reconstructed images.

The post-filtering reduces artifacts (thin lines, circles) as a result of the input image limitations and the discretization of the images (pixels, projections, and intensity). But post-filtering modifies (as well as the intermediate frequency filtering) the information content of the images, which finally affects the feature marking done by the DL models in this work. The original images are noisy, but with known statistical models behind them. The reconstructed images are noisy, too, and although the noise level can be low, the noise is totally different from the X-ray image noise and cannot be described by statistical models. The noise depends on various aspects of the entire reconstruction data flow pipeline. All highly non-linear and complex models, including DL models like SAM and Mask R-CNN, are sensitive to noise with unpredictable output. Moreover, the noise, or distortion, in reconstructed images can contain damage like areas. These artifacts can also result from the measuring process, e.g., by X-ray scattering and the aforementioned polychromatic X-ray energy distribution. Finding very small damages requires a sufficient suppression of the marking of such reconstruction artifacts. Due to the missing ground-truth of real damages, the evaluation of false damage marking with respect to reconstruction artifacts is a challenge.

### 2.2 Geometric analysis of CT slice images

In this work, hidden damages are considered as internal material deformation directly visible in the input data, but not in the measuring data (the projection images). The extent of the global damage determines the visibility of the internal sub-damages in the reconstructed image slices. Due to noise and reconstruction artifacts, not all sub-damages are directly visible by visual inspection.

Edge detection using, e.g., Canny filters, can improve the detection of sub-damages, but still misses an automated geometric characterization. Due to composite layer structures and fiber matrix patterns, edge detectors fail to isolate damages. Simple CNN-based pixel classifiers (Bosse et al., 2024) have a short detection range and cannot distinguish layer boundaries from damages robustly.

Geometric features of impact damages are:

1. Hull of the enclosed damage area (crack, delamination, deformation of layer boundary);
2. Volume of the damage defined by a closed hull surface (crack, delamination);
3. Projection area of the damage volume with respect to a given orthogonal axis;
4. Mass-of-center point of a damage volume;
5. ROI bounding box of a damage, including width, height, and angle.

### 2.3 Synthetic data generation

The main issue with engineering data is the limited parameter space, i.e., restrictions on material variations, composite structures, measuring, and damage parameters. Finally, the missing ground truth of experiments with induced damages is a hard limiting factor for the evaluation and assessment of DL models applied to such a thin data base.

To overcome these limitations, we generate input data synthetically by combining geometric damage modeling and X-ray simulation. In Bosse et al. (2024) we applied this technique for data-driven pore analysis in die-casted aluminum plates by using X-ray radiography images. The basic workflow is:

1. Definition of a simplified damage model (pores were approximated by ellipsoids);
2. Specification or measurement of the damage model parameters with statistical analysis (in the case of pores, these were ellipsoid parameters derived from μCT measurements);
3. Monte Carlo simulation of damage parameter and creation of a large set of synthetic damages;
4. Automatic generation of a CAD model of a host component (e.g., a plate) with damages, typically using a Constructive Solid Geometry model;
5. Transformation of the CAD model into a simulation model, here a triangular material mesh-grid model;
6. Performing the data simulation, hereby using the GVXR X-ray simulation library (Vidal et al., 2024).

In contrast to the previous work in Bosse et al. (2024), we face three significant challenges:

1. Impact damages, especially in composite materials, are highly complex with respect to geometric shapes, extent, and impact energy dependency;
2. Pores are subtractive damages reducing the material density and mass-volume, whereas impact damages deform materials by preserving the mass-volume;
3. In the pore analysis use-case, we applied feature marking models to two-dimensional radiography images (without geometric transformation). In this work, we apply feature marking models to three-dimensional reconstructed image slices as a result of a highly complex mathematical transformation of planar measuring data.

One of the major challenges is the modeling of the constant mass-volume boundary condition if a damage is added to a host material. We have chosen a simple mass-spring model as a surrogate helper model (based on Alijdens, 2023) to satisfy this condition and to provide a simplified impact deformation propagation along the layer axis.

**Figure 1:**
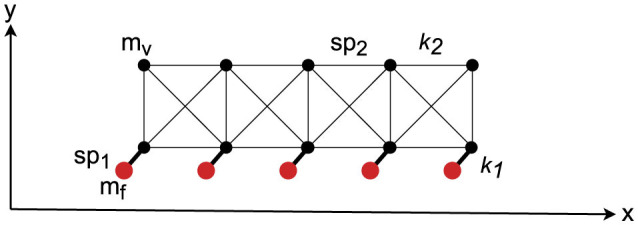
Basic two-dimensional mass-spring mesh-grid model. There are elastic nodes (*m*_*v*_) and fixed nodes (*m*_*f*_), each class connected by springs *sp*_1_ and *sp*_2_ with spring constants *k*_1_ and *k*_2_, respectively. The fixed nodes drive the bottom node row, finally moving the upper node row.

**Figure 2:**
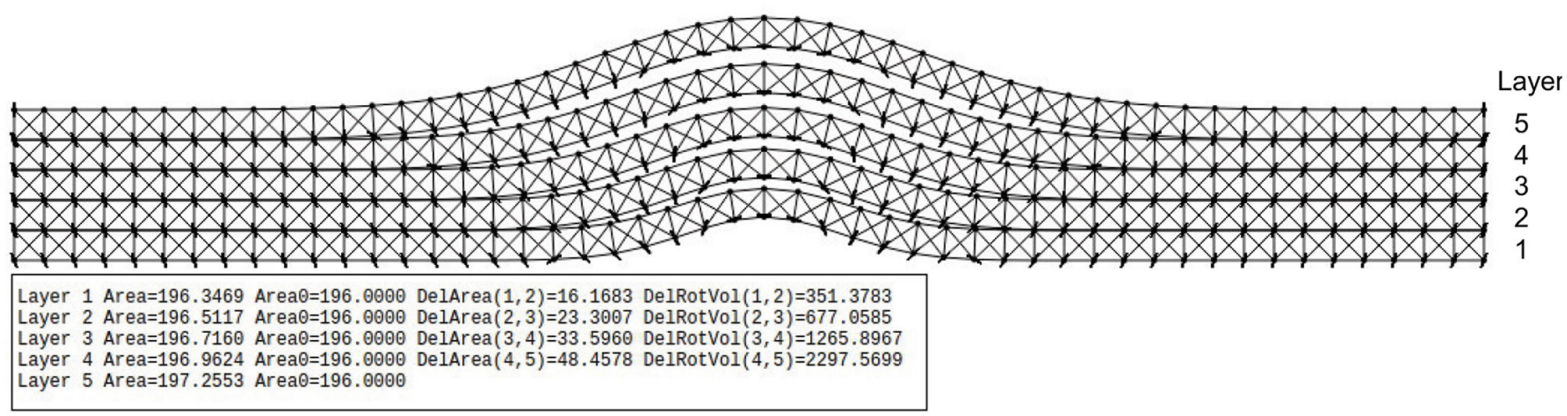
Multi-layer mass-spring mesh-grid model. Each layer is modeled independently. The bottom shape of a layer is given by the driving (invisible) fixed mass nodes *m*_*f*_, with their positions given by the generator function. The table at the bottom shows analysis results from an impacted plate. The *area* and *area0* are cross-sectional layer areas after and before the deformation, which are nearly equal, showing the satisfaction of the constant mass-volume constraint. The example shows a five-layer material with *T*_*x*_ = *T*_*y*_ = 1.2, *S* = 1.5, *D* = 0.005.

Each layer consists of a set of nodes connected by springs. A driving force is applied to the bottom nodes only. The top nodes will be displaced based on the spring forces and elasticity, as shown in Figure 1. The multi-layer model is composed of individual mass-spring layers, as shown in Figure 2. The lower shape of each layer is controlled by the fixed mass positions given by the layer generator functions. Based on experimental μCT investigation, the deformation increases with increasing layer from bottom to top (assuming the impact impulse is applied to the bottom layer). We assume a Gaussian-like deformation due to the impact event described by a parameterizable Gaussian function, which is applied to all layers. Physically, the deformation in composite sandwich plates is much more complex. The deformation of metal layers will be plastic, but with constant mass-volume (no change in material density). The plastic deformation is mainly driven by the mostly elastic deformation of the fiber layers, which swing back partially after the impact energy decreases. If the temporary deformation of the fiber layers was high, then cracks inside the matrix occur, which are not modeled in the synthetic model. Instead, we assume plastic deformation in all layers.

The mathematical generator function *G* for the deformation (driving nodes in the bottom layer of each layer) is given by (with some initial parameter settings used below):


(1) $$\begin{array}{c} W=50,h=2,k=1,k_{1}=100,k_{2}=100,D=2,S=1,\\ T_{x}>1,T_{y}>1\\ G\left(x,x_{0},\sigma \right)=ke^{-\frac{{\left(x-x_{0}\right)}^{2}}{2}\sigma^{2}}\\ L_{i}\left(y_{0},D_{i},S_{i}\right):{\mathbb{R}}^{3}\to {\mathbb{R}}^{W\cdot \left(h+1\right)},i\in \left\{1,2,..\right\},D_{i}=D\cdot T_{x}^{i},S_{i}=S\cdot T_{y}^{i} \end{array}$$

with *W* and *h* as the number of horizontal and vertical points, *K*_*i*_ as the driving an inner spring constants, *T*_*x*_ and *T*_*y*_ as the horizontal and vertical stretching factors, *D* and *S* as the normalized damage width and height. The Gaussian generator function *G* is used by a layer generator function *L*, which generates a point vector defining the positions of the fixed lower bound and free lower and upper bound nodes of the mass-spring model. The relative width and height of the deformation must be monotonically increased, expressed by *T*_*x*_ and *T*_*y*_ parameters, otherwise an upper layer collides with a lower layer. The unified mass of each node was set to 0.001 (arbitrary units), which is not relevant for the stationary state (gravity is not considered). The fixed nodes are assigned to infinity mass. The basic algorithm for the layer generation function, creating mass nodes and the springs, is shown in Alg. 1 in App. A.

This model is still oversimplified as it can be seen from the μCT results of real samples presented in this work. But this simplified model can be used to evaluate and calibrate the feature marking models (considered as a gold standard) because the geometric damage characteristics can be calculated from the numerical material model.

The damages are characterized by the delamination between two-layer boundaries (the gap). Firstly, the slice area (in *x*-*y* axis directions) *A*_*xy*_ is calculated using all upper node points from layer *l*_*i*_ and all lower node points from layer *l*_*i*+1_, forming a closed polygon. The slice area *A*_*xy*_ is then used to calculate a rotated volume *V* around the *z* axis, assuming that the closed polygon consists of *n* points with two identical start and end points:


(2) $$\begin{array}{l} \;A_{xy}=\left|\overset{n-1}{\underset{i=1}{\sum }}\left(\frac{x\left(p_{i}\right)\cdot y\left(p_{i+1}\right)}{2}-\frac{x\left(p_{i+1}\right)\cdot y\left(p_{i}\right)}{2}\right)\right|\\ x_{c}=\overset{n-1}{\underset{i=1}{\sum }}\left(x\left(p_{i}\right)+x\left(p_{i+1}\right)\right)\cdot \left(x\left(p_{i}\right)\cdot y\left(p_{i+1}\right)-y\left(p_{i}\right)\cdot x\left(p_{i+1}\right)\right)\\ y_{c}=\overset{n-1}{\underset{i=1}{\sum }}\left(y\left(p_{i}\right)+y\left(p_{i+1}\right)\right)\cdot \left(y\left(p_{i}\right)\cdot x\left(p_{i+1}\right)-x\left(p_{i}\right)\cdot y\left(p_{i+1}\right)\right)\\ \;\alpha =\sqrt{{\left(\frac{x_{c}}{6A}\right)}^{2}+{\left(\frac{y_{c}}{6A}\right)}^{2}}\\ \;V=2\pi A_{xy}\alpha \end{array}$$

The *x*_*c*_ and *y*_*c*_ values are the centroid coordinates of the rotated damage volume.

### 2.4 Mask R-CNN

Mask R-CNN combines the elements of object detection (producing a bounding box around each localized object) and semantic segmentation (assigning a class label to each pixel in the image). The Mask R-CNN model is an extension of the Faster R-CNN, which is a powerful baseline system. It enhances Faster R-CNN by adding a branch to predict segmentation masks for each RoI in parallel to the existing branch for classification and bounding box regression. The mask generation branch is a simple, fully convolutional network (FCN). Mask R-CNN uses the innovative quantization-free RoIAlign layer instead of the RoIPool used in Faster R-CNN, which performs coarse spatial quantization for feature extraction. This allows for more precise mask predictions without misalignment. The model predicts a binary mask independently for each class without any competition between them, separating the classification task performed by the network's RoI classification branch from the mask generation task. This contrasts with the semantic segmentation approach proposed by Long et al. (2015), where segmentation and classification are coupled (Ren et al., 2015; Girshick, 2015; He et al., 2017).

**Figure 3:**
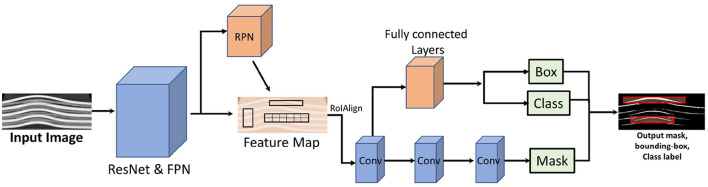
Architecture of Mask R-CNN. The network consists of a backbone network (ResNet50 + FPN), Region Proposal Network (RPN), RoIAlign, and two parallel heads for mask generation and bounding box prediction/classification. Image adapted from Podder et al. (2021).

The architecture of Mask R-CNN as shown in Figure 3 can be divided into several key components:

1. **Backbone network:** Typically a ResNet or ResNeXt, used for feature extraction. This network processes the input image to generate a convolutional feature map. In this paper, we use a combination of ResNet with 50 layers and a Feature Pyramid Network (FPN) as the backbone. FPN addresses the challenge of multi-scale object detection by creating a pyramid of features, making it effective for tasks involving objects of various sizes.
2. **Region proposal network (RPN):** A fully convolutional network that generates candidate object proposals. It slides a small network over the convolutional feature map and, at each location, simultaneously predicts objectness scores (likelihood of an object) and bounding box coordinates.
3. **RoI align:** Mask R-CNN improves upon the RoI Pooling used in Faster R-CNN by introducing RoI Align. This method ensures that the regions of interest (RoIs) are accurately extracted from the feature map without any misalignment, preserving spatial coherence by using bilinear interpolation to avoid quantization errors.
4. **Bounding box head:** For each RoI, a small fully connected network predicts the class of the object and refines the bounding box coordinates (bounding box regression).
5. **Mask Head:** In parallel with the bounding box head, the mask head generates a binary mask for each RoI. This head is typically a small convolutional network that produces a segmentation mask for each object class.

The loss function (*L*) in Mask R-CNN is composed of three components, classification loss (*L*_cls_), Bounding box loss (*L*_box_), and Mask loss (*L*_mask_):


$$L=L_{\text{cls}}+L_{\text{box}}+L_{\text{mask}}$$

Each term in the equation shown above corresponds to a specific task within the model:

1. **Classification loss**:

It uses a standard cross-entropy loss (log loss) to measure the error between the predicted class probabilities and the true class labels. If *p*_*i*_ is the predicted probability of the *i*-th class (where *i* ranges from 1 to the number of classes), and *t* is the true class label, the classification loss *L*_cls_ is given by:


$$L_{\text{cls}}=-\log\left(p_{t}\right)$$

2. **Bounding box regression loss**:

This component refines the coordinates of the bounding boxes predicted by the network. It uses a smooth L1 loss to measure the difference between the predicted and true bounding box coordinates. Let *v* be the true bounding box coordinates and $\hat{v}$ be the predicted coordinates. The bounding box regression loss *L*_box_ is:


$$L_{\text{box}}={\text{smooth}}_{L1}\left(v-\hat{v}\right)$$

The smooth L1 loss is defined as:


$${\text{smooth}}_{L1}(x)=\{\begin{array}{ll} 0.5x^{2} & \text{if }|x|<1\\ |x|-0.5 & \text{otherwise} \end{array}$$

3. **Mask loss**:

The mask loss is computed using a pixel-wise binary cross-entropy loss. It is used to measure the accuracy of the predicted binary masks for each class. For each RoI, Mask R-CNN predicts a binary mask for each class, but only the mask corresponding to the ground-truth class is used in the loss calculation. Let *m* be the true binary mask and $\hat{m}$ be the predicted mask. The mask loss *L*_mask_ for a given RoI is:


$$L_{\text{mask}}=-\underset{p,q}{\sum }\left[m_{pq}\log\left({\hat{m}}_{pq}\right)+\left(1-m_{pq}\right)\log\left(1-{\hat{m}}_{pq}\right)\right]$$

Here, *p* and *q* index the pixels in the mask.

### 2.5 Segment anything model

The Segment Anything Model (SAM) features several key capabilities. It is designed for promptable segmentation tasks, allowing it to produce valid segmentation masks based on various prompts such as points, boxes, and text descriptions. SAM can compute masks in real-time, making it ideal for applications that require rapid object segmentation, like autonomous driving and robotics. It excels in zero-shot performance, effectively handling diverse segmentation tasks with minimal prompt engineering. Additionally, SAM is aware of object ambiguity, enabling it to generate masks even for partially occluded or overlapping objects.

**Figure 4:**
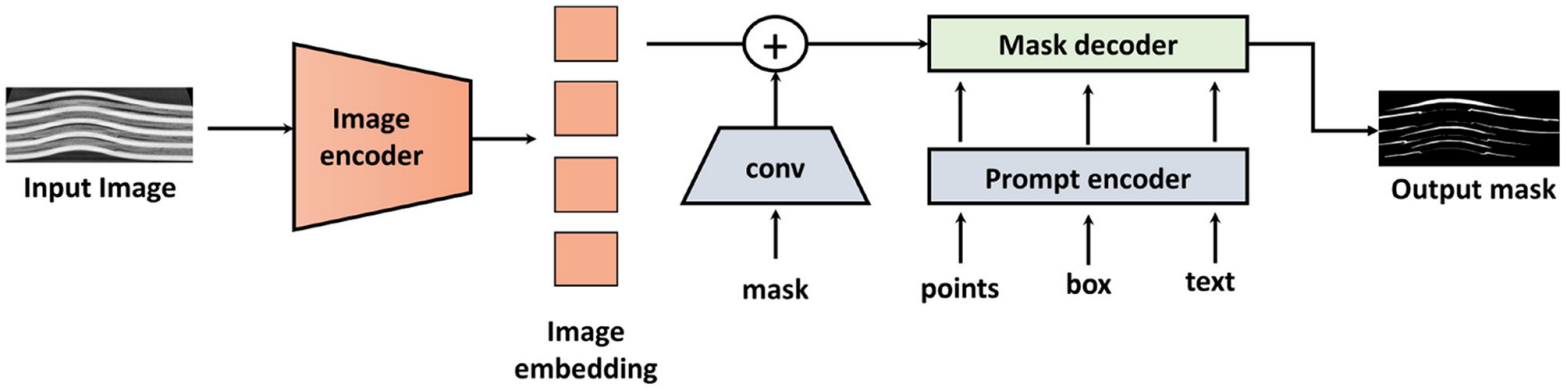
Architecture of SAM (Segment Anything Model). The architecture is divided into three main components: Image Encoder, Prompt Encoder, and Mask Decoder. Image adapted from Kirillov et al. (2023)

SAM's architecture, as shown in Figure 4 is divided into three main components:

1. **Image encoder**: This component processes the input image to generate image embeddings. SAM uses a Vision Transformer (ViT) as its backbone to create these image embeddings. Currently, three models, namely ViT-H, ViT-L, and ViT-B, are available. The ViT splits the input image into patches, processes these patches using self-attention mechanisms, and generates a global feature representation of the image. These feature representations contain spatial and contextual information of the image.
2. **Prompt encoder**: This component processes two sets of prompts: sparse prompts (points, boxes, text) and dense prompts (masks). The points and boxes are encoded using positional encodings, which are summed up with the learned embeddings for each prompt type. Convolutions are used to embed the mask prompts, which are then summed element-wise with the image embedding. The text prompts are optional and not always included. They are encoded using a text encoder based on a transformer-based language model.
3. **Mask decoder**: This component produces the segmentation masks by processing the image and prompt embeddings. It maps these embeddings and an output token to the mask. It uses a modification of the Transformer decoder block followed by a dynamic mask prediction head (Buhl, 2024).

The loss function used in the SAM is a combination of Dice loss and Cross-Entropy loss. Both the losses are computed separately, and the weighted sum of both results in the final loss function called the DiceCELoss implemented via MONAI library (Cardoso et al., 2022). The loss function is defined as follows:


(3) $$\begin{array}{l} L=\alpha L_{\text{Dice}}+\beta L_{\text{CE}} \end{array}$$

where $L_{\text{Dice}}$ is the Dice loss, $L_{\text{CE}}$ is the Cross-Entropy loss, and α and β are the weighting factors for the Dice and Cross-Entropy losses, respectively. The Dice loss is defined as (Milletari et al., 2016):


(4) $$\begin{array}{l} L_{\text{Dice}}=1-\frac{2\sum_{i}p_{i}g_{i}}{\sum_{i}p_{i}^{2}+\sum_{i}g_{i}^{2}} \end{array}$$

where *p*_*i*_ are the predicted probability and *g*_*i*_ are the ground truth binary label for each pixel *i*.

The Cross-Entropy loss for binary classification in image segmentation is defined as:


(5) $$\begin{array}{l} L_{\text{CE}}=-\frac{1}{N}\underset{i}{\sum }\left[g_{i}\log\left(p_{i}\right)+\left(1-g_{i}\right)\log\left(1-p_{i}\right)\right] \end{array}$$

where *p*_*i*_ is the predicted probability and *g*_*i*_ is the ground truth binary label for each pixel *i*, and N is the total number of pixels in the image.

The DiceCE loss balances the complementary biases of the CE and Dice loss. On one hand, the CE encourages the predicted region to be in similar proportions to that of the ground truth, Dice loss favors better prediction of small structures. It results in a more effective loss function that can handle both overall class balance and smaller important features in the segmentation task.

### 2.6 Model explainability

The Detectron2 and SAM are powerful DL models for the segmentation tasks. But like other DL models, they are also very opaque in terms of their decision-making processes. That is why such models are rightly labeled as “black boxes.” Explainable Artificial Intelligence (XAI) has emerged as a crucial tool that addresses this lack of transparency. It helps in making the inner workings and decisions that the deep learning models are making more interpretable. The interpretability (or explainability) refers to the easy understanding of the model results by humans. The terms interpretability and explainability are used interchangeably in this paper, as is the case in most of the XAI literature (Gipiškis et al., 2024). The automated damage segmentation in CT slices of FML material poses a unique challenge due to the varied damage morphologies, subtle contrast variation caused by varied densities in the prepreg layer, model biases in damage feature segmentation, and intricate layer interfaces. These challenges demand the need to understand how the model perceives the damage feature.

The Integrated Gradients (IG) method proposed by Sundararajan et al. (2017) is a widely used XAI technique that attributes the model output to its input features, thus improving the model's interpretability. It produces an intuitive attribution map highlighting the most influential regions in the CT slices that are responsible for the damage segmentation. The attribution map assigns attribution scores (a high attribution score means high importance for the input feature) to all the pixels in the input image. Therefore, making it easier to identify the critical parts of the images and to qualitatively compare the results from different models. Moreover, the IG is model-agnostic, meaning that it is applicable across all neural network architectures. The IG formula along the *i*^*th*^ input dimension is shown in Equation 6 where the function *F* represents a deep neural network, *x* is the input to the network, and *x*′ be the baseline input image. The baseline could be a Gaussian noise image or a completely black or white image. A good baseline will have a zero or near-zero attribution score for all the pixels in the baseline image.


(6) $$\begin{array}{l} {\text{IntegratedGrads}}_{i}\left(x\right)::=\left(x_{i}-x_{i}^{\prime }\right)\times \int_{\alpha =0}^{1}\frac{\partial F\left(x^{\prime }+\alpha \times \left(x-x^{\prime }\right)\right)}{\partial x_{i}}d\alpha \end{array}$$

A straight line path from the baseline *x*′ to the input *x* is considered, and the gradients are computed at all points along the path. IG is calculated by cumulating (by path integral in the formula) these gradients. The integral in the IG calculation is approximated via a summation given in Equation 7 where *m* is the number of steps in the Riemann approximation of the integral.


(7) $$\begin{array}{l} \;{\text{IntegratedGrads}}_{i}^{\text{approx}}\left(x\right)::=\\ \left(x_{i}-x_{i}^{\prime }\right)\times \sum_{k=1}^{m}\frac{\partial F\left(x^{\prime }+\frac{k}{m}\times \left(x-x^{\prime }\right)\right)}{\partial x_{i}}\times \frac{1}{m} \end{array}$$

## 3 Methodology

This work aims to investigate automated image-based damage diagnostics. The problem can be defined and summarized as follows:

1. Input data is a sliced image set of material-density images showing the cross-section of the specimen. A three-dimensional data set volume *V* consists of *n* image slices of size *p* × *q* pixels. The intensity (an integer value with an 8-16 Bits range) of each pixel is related to the material density; zero (or minimum) is related to air or vacuum (no material), the highest intensity value is related to the material with the highest density (here aluminum). Damages are regions with lowered density. Each pixel (or voxel in three dimensions, including the slice axis) has a specific spatial resolution (here about 18 μm) determined by the measuring system and the CT algorithms used to reconstruct the slice stack from radial projection images.
2. An intermediate output are overlay image with the same size as the original input images (*p* × *q* pixels). The overlay images mark regions of interest (damage candidates). The overlay images can be the direct output of the DL models considered in this work or can be created indirectly and based on aggregate data (ROI) from the DL models. A rough approximation of an ROI is a rectangle, with improved spatial accuracy by using a polygon.
3. The ROI candidate markings as the output from the DL models are transferred in a linear set of pixel coordinate lists, either one list for each slice image or one list for the entire slice volume.
4. The linear pixel coordinate lists are clustered into groups of pixel lists. Each group should mark one sub-damage which must be separable from other sub-damages, either geometrically (no overlapping regions) or by damage class (crack vs. delamination, characterized by both low pixel intensities, but different geometric shapes and surrounding). For further geometric damage characterization, hull approximations can be applied. Two-dimensional clustering results often result in high inaccuracy due to image noise, which can be improved by three-dimensional clustering correlating voxels instead of pixels.
5. The groups are geometrically characterized and analyzed, finally collected in a low-dimensional damage table.

**Figure 5:**
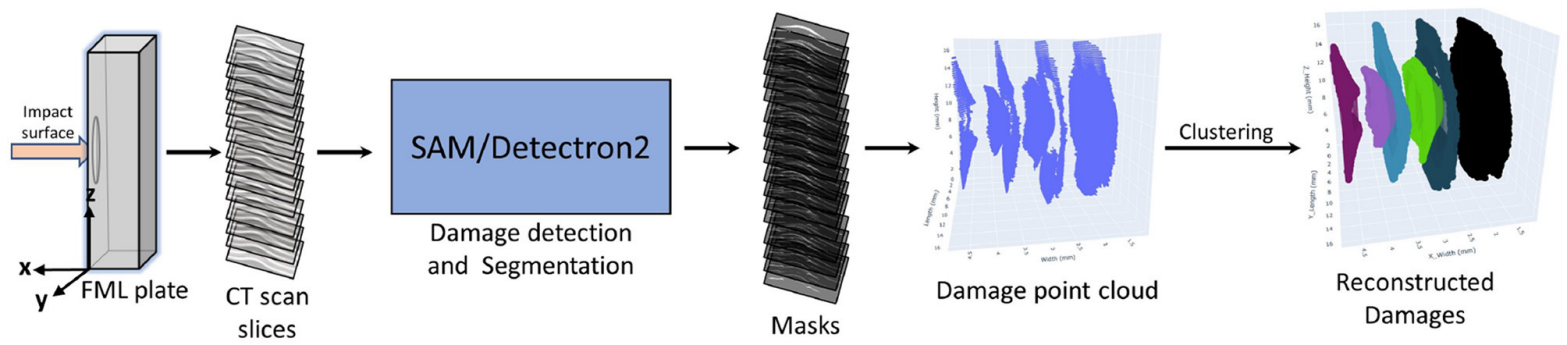
Process pipeline for the damage detection, segmentation, and characterization within the FML plate.

The X-ray CT datasets (DL model training data) for each specimen consist of 1,024 slices, where each slice represents a cross-section in the X-Y plane at various heights along the z-direction as shown in the Figure 5. Each slice contains several damage features, along with some artifacts and noise. These datasets (CT scan slices) for each energy level were passed through the deep learning pipeline one by one. The input is a 300 × 950 pixels 8-bit grayscale image having pixel values between 0 to 255, and the output is a binary image of the 300 × 950 pixels. The generated output damage segmentation masks were then stacked over one another, similar to the CT scan slices. This stack of segmented damage masks was used to generate the damage point cloud. The point clouds contain the marked coordinates of the damage in three dimensions. A clustering algorithm called DBSCAN (Density-Based Spatial Clustering of Applications with Noise) was then applied to this point cloud to form clusters of the damages. The three-dimensional point clouds are output as an HTML file. A concave hull algorithm based on alpha shape was used to calculate the volumes and projected areas of these clusters, enabling detailed characterization of the damage attributes.

The quality of the masks determines the quality of the reconstructed damages and their characterization. The performance of the deep learning models, Segment Anything Model (SAM) and Detectron2, was evaluated using Intersection over Union (IoU) and F1-score. IoU and F1-score are the commonly used metrics in object detection and segmentation tasks. The formula for IoU is given by:


(8) $$\begin{array}{l} IoU=\frac{\text{area}\left(P_{d}\cap G_{d}\right)}{\text{area}\left(P_{d}\cup G_{d}\right)} \end{array}$$

where *P*_*d*_ and *G*_*d*_ are the predicted damages and the ground-truth damages, respectively (Pham et al., 2020).

The formula for the Precision, Recall, and F1-score is calculated using True Positives (TP), False Positives (FP), and False Negatives (FN).


(9) $$\begin{array}{l} \text{Precision}=\frac{\text{TP}}{\text{TP}+\text{FP}} \end{array}$$

(10) $$\begin{array}{l} \text{Recall}=\frac{\text{TP}}{\text{TP}+\text{FN}} \end{array}$$

(11) $$\begin{array}{l} \text{F1}=\frac{2\cdot \text{Precision}\cdot \text{Recall}}{\text{Precision}+\text{Recall}} \end{array}$$

The performance of the deep learning models also greatly improves with the preprocessing of input images. The pre-processing helps in enhancing the images in such a way that the damage features are easier to extract and learned. The preprocessing steps included the normalization of images, noise removal by a Gaussian filter, and contrast enhancement. A Gaussian filter was applied, as the primary noise source follows a Gaussian distribution in X-ray images (Lee and Kang, 2021). For contrast enhancement, the Contrast Limited Adaptive Histogram Equalization (CLAHE) algorithm was used. CLAHE is a widely used technique in image processing that improves contrast by adjusting the intensity of pixel values. The key steps involved are:

1. Histogram computation: the histogram of pixel intensities within the image is computed, representing the distribution of intensity values across the image.
2. Adaptive partitioning: the image is divided into small overlapping tiles or patches. The size of each tile is chosen to be small enough to capture local variations in intensity effectively.
3. Histogram equalization within tiles: histogram equalization is independently applied to each tile, enhancing the contrast within each tile by stretching the intensity range, thereby improving local contrast.
4. Contrast limiting: to prevent overamplification of noise in regions with low local contrast, contrast enhancement is limited by clipping the cumulative histogram within each tile.
5. Interpolation: the contrast-enhanced tiles are combined to reconstruct the final enhanced image, involving interpolation or blending of neighboring tiles to ensure smooth transitions between regions.

The damaged areas in the preprocessed slices were hand annotated in COCO format using an online annotation tool (Skalski, 2019). For object detection and picture segmentation tasks, the COCO format is a popular data format that arranges annotations in JSON files with information such as bounding boxes, segmentation masks, and categories. It is designed for the easy interoperability between different frameworks, tools in computer vision. The annotations were downloaded as a JSON file and used to create damage masks. The annotation file, along with the processed images, was passed into the Detectron2 for the training and evaluation. The SAM uses the processed images and corresponding masks for the training and evaluation.

**Figure 6:**
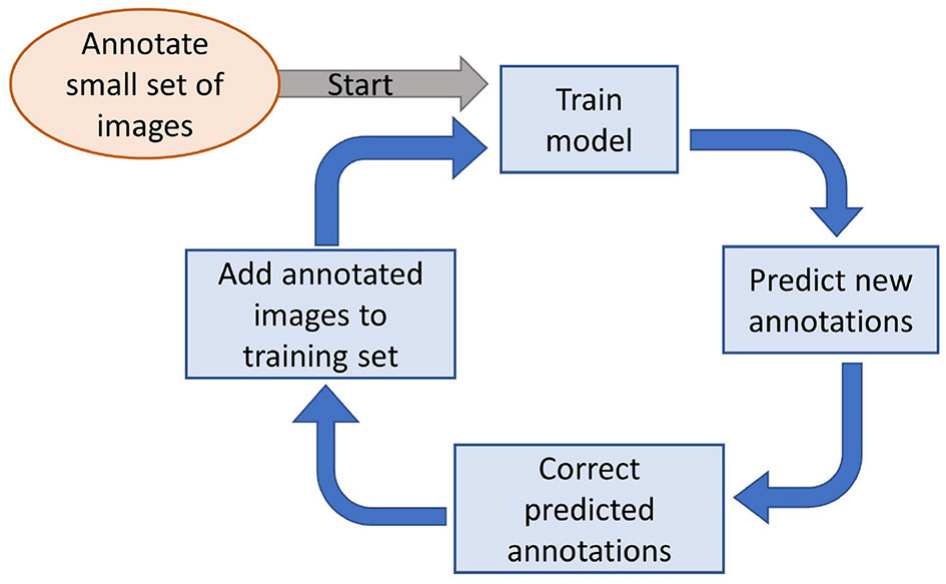
Cyclical process to fasten the annotation process of the training images.

Manual annotation of images is a time-consuming process. To speed up the generation of annotated training data, the iterative process outlined in Figure 6 was employed. Kirillov et al. (2023) used a similar method for faster annotation of their training images. The process begins with a small set of annotated images used to train the initial deep learning model. If the model's performance is unsatisfactory, the trained model is then leveraged to generate annotations on additional images. These newly annotated images are reviewed for manual corrections and are combined with the original set to retrain the model. While incorrect annotations still need manual correction, this approach significantly reduces the time required for annotation, as many of the model-generated annotations might often be accurate. There are some challenges involved in annotating the images using this process. The error in annotations is propagated if adequate corrections are not made to the predicted annotations by the model. So the predicted annotations must be thoroughly reviewed and corrected by domain experts. If the errors are left uncorrected, they will be passed on to the model for training. The model will treat the incorrect annotations as the ground truth, and thus errors will amplify in the subsequent iterations. Additionally, bias may also be introduced in the training dataset due to human annotator bias. Subjective biases may be carried over during the iterative process of annotation. If the initial small training dataset is not diverse enough, then the model may learn a biased representation. The methodological limitations inherent in the iterative annotation process are resolved by implementing specific measures. In order to minimize the risk of error propagation, the model-generated annotations are subjected to a thorough review by domain experts. These experts meticulously correct inaccuracies in the annotations manually before adding them to the training dataset. It ensures that the ground truth remains reliable. Therefore, it prevents the amplification of errors in subsequent training iterations. In order to counter the potential human annotator bias, the initial annotated dataset was designed to be as diverse as possible. Thus, it represents various scenarios and edge cases within the domain. This is a critical step, making sure the model learns a balanced representation and reduces the risk of biased predictions during subsequent annotation cycles. By integrating these safeguards in the annotation process, the reliability and efficiency of the annotation process are improved.

The SAM model used for the training is ViT-B (sam-vit-base), which is a lighter and faster version compared to the other two available options (ViT-H, ViT-L). The model was fed with the bounding box around the damages as well for the training, and the point grid was used as the prompt for the inference, as the bounding box is not known during the inference. The model used in the implementation of Detectron2 is ResNet-50-FPN-3x (R50-FPN), which is well-suited for damage detection and segmentation tasks. Pretrained on the COCO dataset, this model demonstrates efficient training and inference times while achieving relatively high box and mask Average Precision (AP) (Rani et al., 2024). The Mask R-CNN model in Detectron2 was trained using the same training images as SAM. Unlike SAM, Detectron2 does not require prompts. The key hyperparameters used for the training and inference of both models are as follows: for the SAM, the optimizer used was Adam with a learning rate of 1 × 10^−5^ and no weight decay, a batch size of 2, and trained for 500 epochs, with a patch size and step size of 256 pixels. Additionally, an inference threshold of 0.95 was used. For the Detectron2, the optimizer used was SGD with momentum, with a learning rate of 2.5 × 10^−4^, momentum of 0.9, and a batch size of 2, with an ROI batch size per image set to 256. The number of workers was set to 2, and the inference threshold was 0.60. The system specifications used are as follows: the CPU is an Intel^®^ Core™ i5-13500H with 12 physical cores and a total of 16 cores, running at a maximum frequency of 2,600 MHz. The total available RAM is 31.73 GB. The GPU used is an NVIDIA GeForce RTX 4060 Laptop GPU with 8,188 MB of total memory and driver version 546.83. The PyTorch version used is 2.2.0+cu121.

## 4 Results and discussion

**Figure 7:**
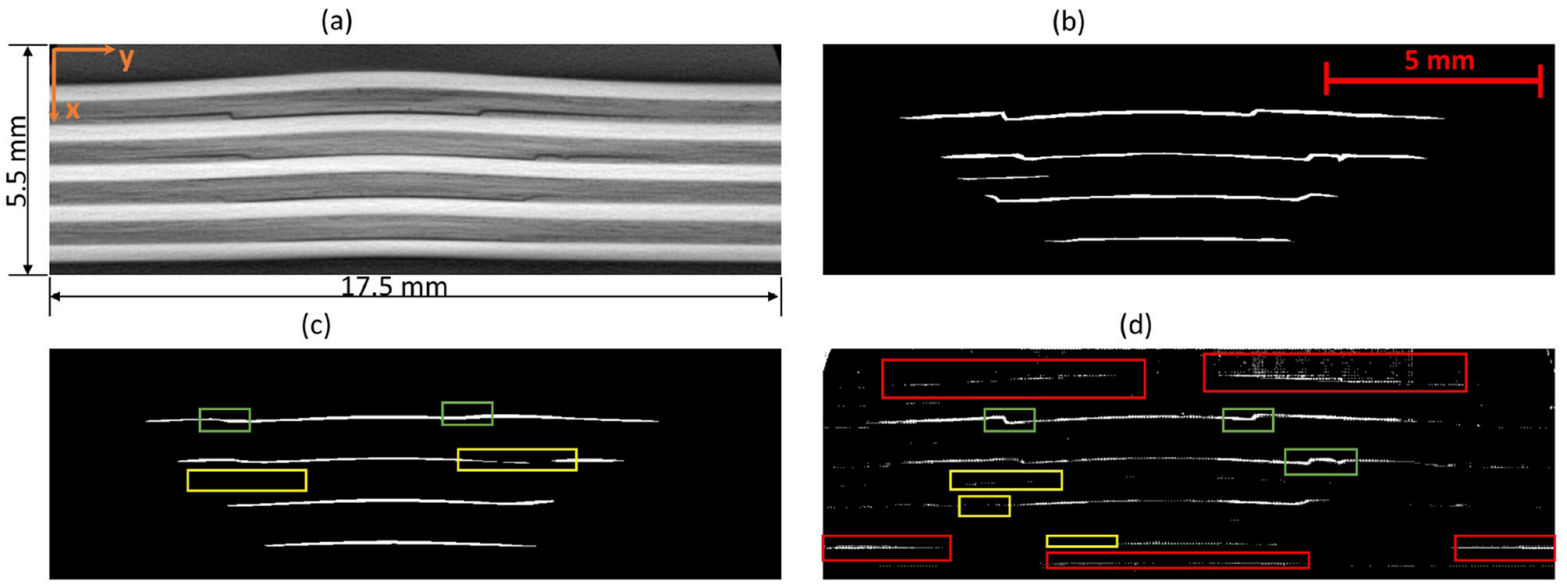
An example CT slice of 7.5J impact energy with damage masks. Red boxes show false positives by SAM, yellow boxes show false negatives by both models, and green boxes highlight SAM's superior performance over Detectron2 in learning complex damage features. **(a)** CT slice for 7.5J. **(b)** Mask-Hand-annotation. **(c)** Mask-Detectron2. **(d)** Mask-SAM.

**Figure 8:**
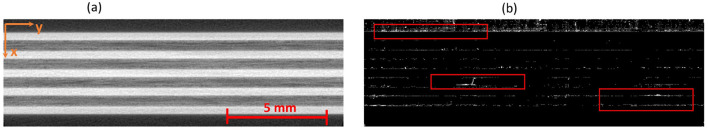
**(a)** An example CT slice of the undamaged plate. Ideally, there should be no damage predictions (white pixels) at all. **(b)** The SAM produces a lot of false positives (indicated by red boxes) whereas the detectron2 produces zero false positives.

The Detectron2 model and SAM were trained on the 28 training images of size 300 × 950 pixels (with 121 unique features) and 8 images (with 35 distinctive features) each of size 300 × 950 pixels are used for the validation. The training and validation losses were tracked, and the training was stopped using an early-stopping technique when the validation loss started to increase to prevent overfitting. The average training time for SAM was observed to be 3 seconds per iteration, whereas it was 0.36 seconds per iteration for the Detectron2. So the Detectron2 trains approximately 8 times faster than the SAM. The time taken to make an inference on a 300 × 950 pixels image by SAM was, on average, approximately 14.55 seconds, whereas for the Detectron2 it was approximately 0.18 seconds. That means Detectron2 is approximately 80 times faster than the SAM. The Detectron2 and SAM were compared for the segmentation of the damages using the performance metric IoU (Intersection over Union), Precision, Recall, and F1 score, each accompanied by their standard deviations calculated by performing the experiments five times, to reflect the consistency of the results as shown in the Table 1. Since there was no absolute ground truth available, domain expert knowledge was used to identify and annotate the damages in the CT slices, referred to as the ground-truth (GT) damages in the whole paper. The Detectron2 with an IoU of 0.53 (± 0.02) shows a moderate overlap between the predicted and ground truth damages, which indicates that it has a reasonable ability to segment the damages. In contrast, the SAM has a low IoU of 0.19 (± 0.01), which indicates that it struggles to accurately segment the damages. The high precision score of Detectron2 [0.77 (± 0.02)] as compared to the SAM [0.31 (± 0.07)] indicates that the majority of the segments identified are actually correct, with a few false positives. The SAM has high false positive predictions as shown in an example prediction Figures 7, 8. To reduce the false positives in SAM, various threshold values were tried in order to improve the F1 score and IoU score. The threshold value of 0.95 was observed to have the best F1 score and IoU value. To get an idea of the number of false positives produced by the SAM and Detectron2, the predictions on the undamaged plates were made as shown in an example Figure 8. The SAM predicted a lot of false positives (damages) in a totally undamaged plate, but the Detectron2, on the other hand, predicted zero false positives (damages). However, since the precision score for the Detectron2 is not one in damaged plates, that indicates that the false positive predictions are coming due to the damaged regions of the plates. On close observation, it can be noticed that the model sometimes overestimates the damage region. The high recall value [0.64 (± 0.02)] suggests low false negative predictions by Detectron2 as compared to the SAM [0.40 (± 0.11)]. Some examples of the false negative predictions by the Detectron2 and SAM are marked by the yellow box in Figure 7. It was observed that the recall value was significantly higher at the lower threshold values as compared to the higher threshold values for the SAM; however, the precision score, IoU, and F1 score deteriorated significantly.

**Table 1:** Performance comparison of Detectron2 and SAM across various metrics for the segmentation of damages within the CT data of FML plates.

**Metric**	**Detectron2**	**SAM**	**SAM (with filter)**
IoU	0.53 (± 0.02)	0.19 (± 0.01)	0.25 (± 0.03)
Precision	0.77 (± 0.02)	0.31 (± 0.07)	0.39 (± 0.14)
Recall	0.64 (± 0.02)	0.40 (± 0.11)	0.55 (± 0.12)
F1	0.70 (± 0.03)	0.35 (± 0.17)	0.46 (± 0.20)

The values represent the mean performance scores (± standard deviation), computed from five repeated experiments for each model.

**Figure 9:**
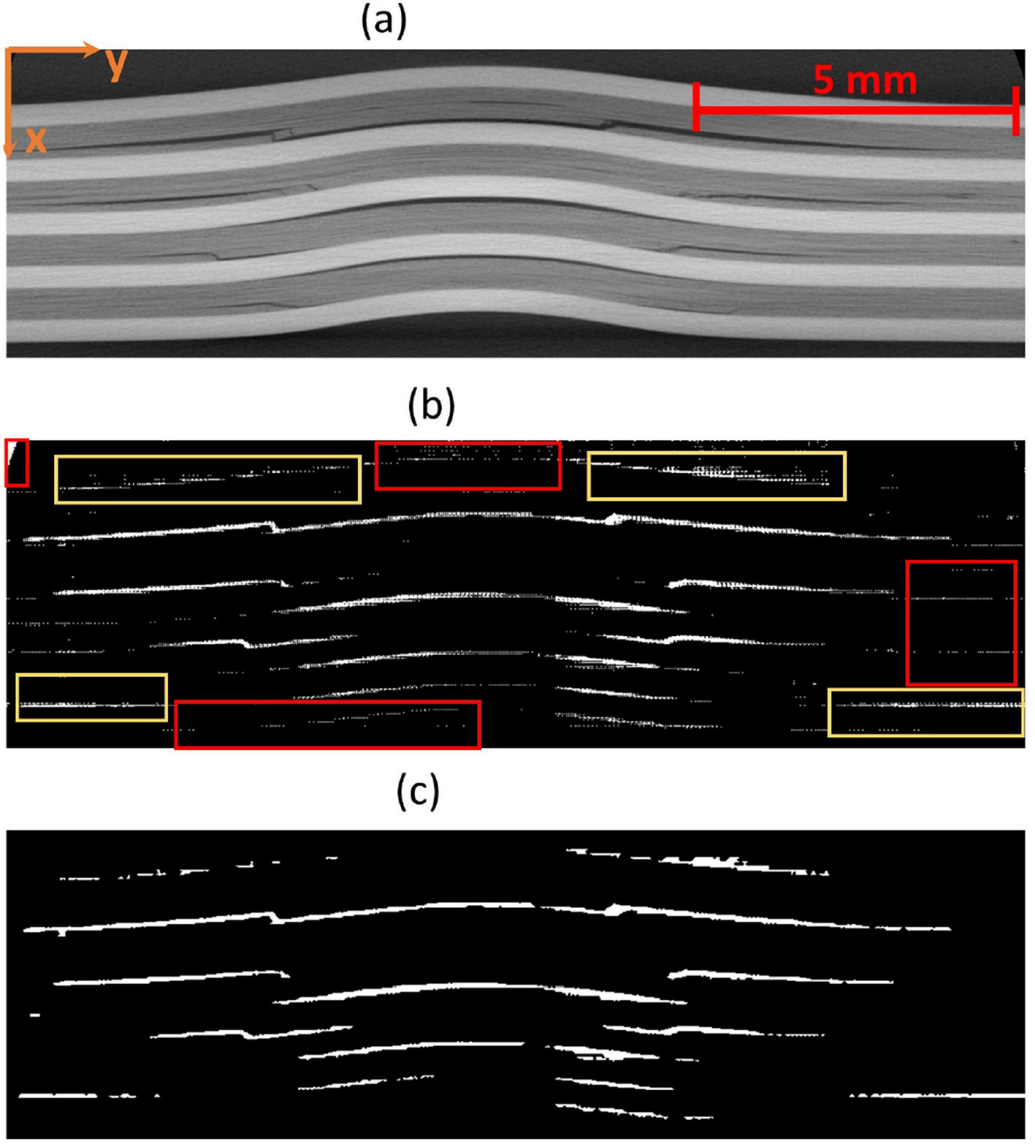
**(a)** An example CT slice of energy level 7.5J. **(b)** The mask produced by SAM generates a lot of false positives some examples are marked by red boxes. **(c)** The filter removes many of those false positives but still, some could not be removed as marked by the yellow box.

To further improve the performance scores in the case of the SAM, the predicted masks are passed through a filter. The high-level algorithm for the filter is shown in Algorithm 1. The filtered masks show significant improvement in all the performance metrics, as shown in Table 1. The improvement in the IoU, precision, recall, and F1 scores is 31.58%, 25.81%, 37.50%, and 31.43%, respectively. However, the standard deviations also increase, indicating less consistency in the scores. The algorithm marks some of the missing false negatives while removing many false positives. An example result is shown in the Figure 9 with the red boxes marking some of the filtered out false positives and yellow boxes marking the unfiltered false positives. However, the performance scores of the Detectron2 still remain higher compared to the SAM with the filter. The F1-score of 0.70 (± 0.03) proves that the Detectron2 has a reliable performance in detecting and segmenting the damage areas, whereas the SAM (with the necessary filtering), with a score of 0.46 (± 0.20), shows its overall poor performance in damage identification and segmentation. These results suggest that the Detectron2 outperforms SAM in all evaluated metrics significantly. The standard deviations of the performance metric scores suggest that the SAM performance is also less reliable as compared to that of the Detectron2. However, the SAM was able to identify the complex damage features better than the Detectron2, as highlighted by green boxes in the Figure 7.

**Algorithm 1:**
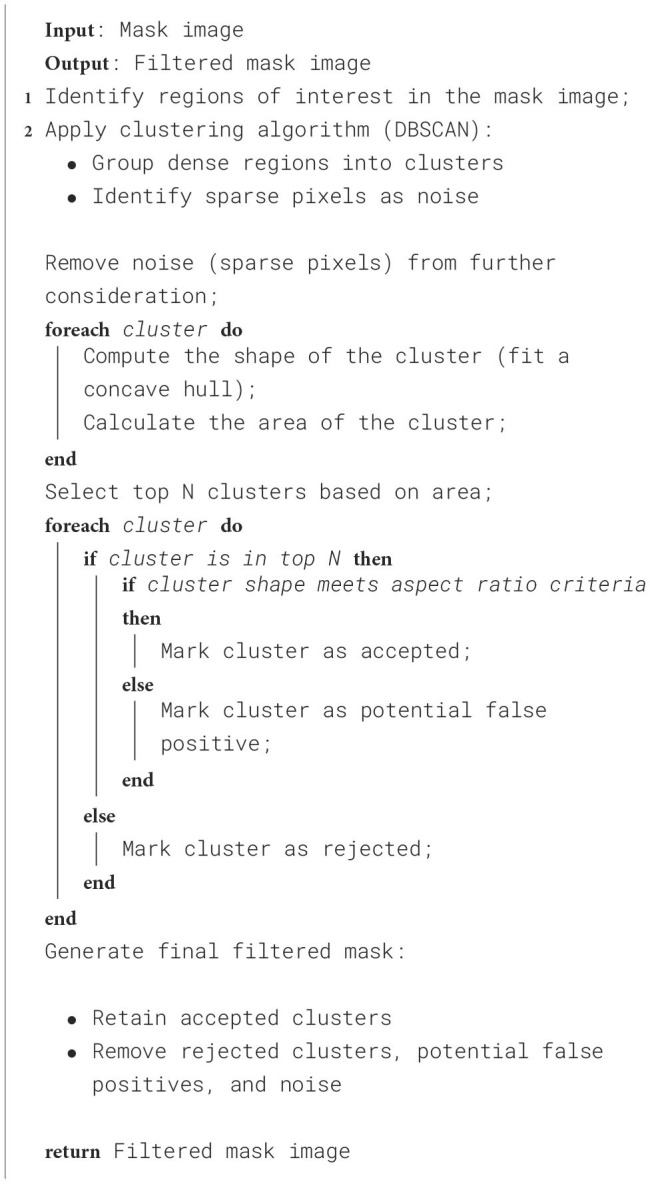
High-level mask filtering algorithm applied on the masks generated by SAM.

**Figure 10:**
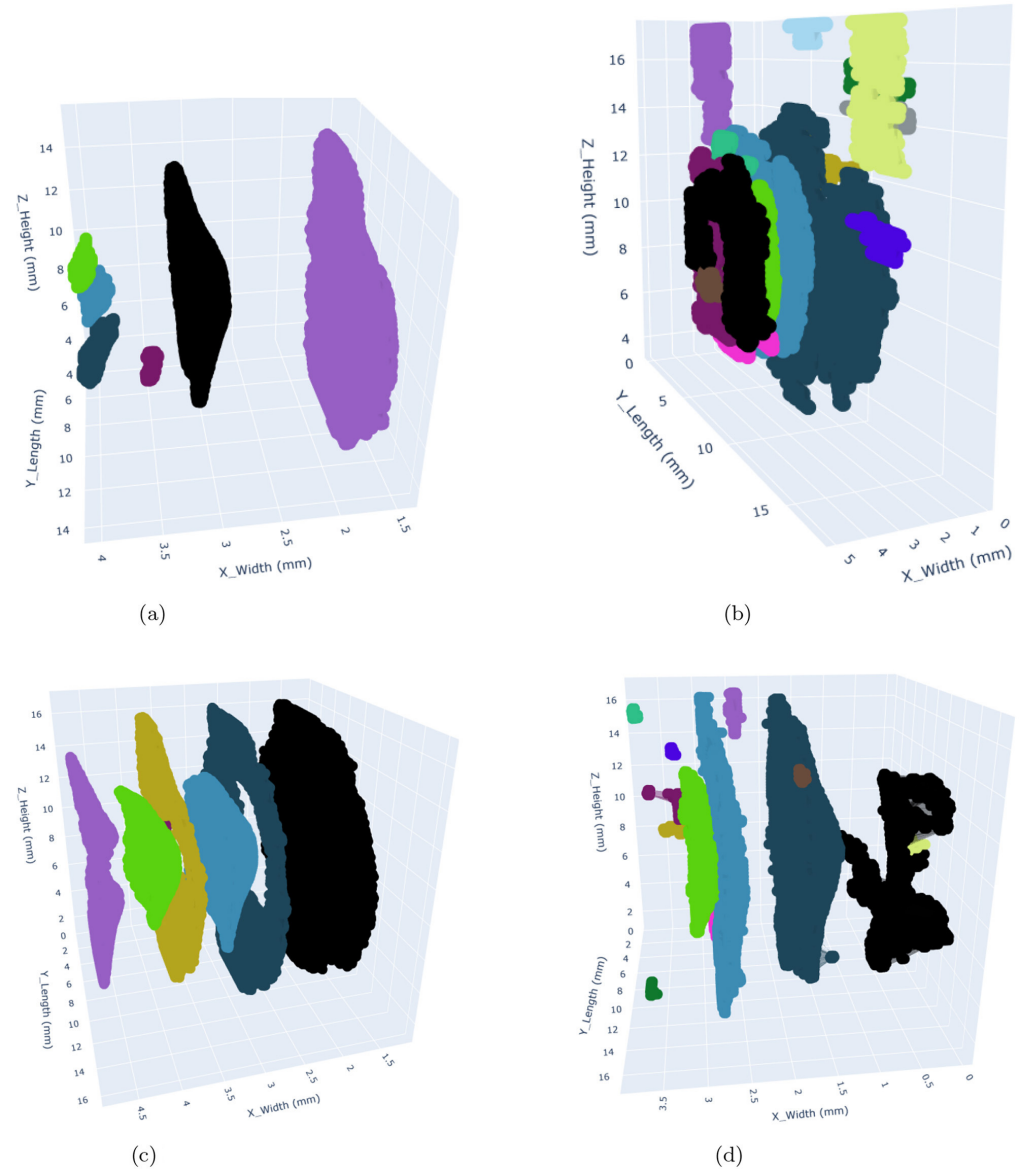
3D visualization of damages within the FML plates occuring at 5J and 7.5J impact energy levels using Detectron2 (left) and SAM (right). The clusters with differences in shape and size highlight the segmentation capabilities of both models. The colors used to represent the clusters are arbitrary and only used to distinguish different clusters from each other. **(a)** Detectron2 - 5J. **(b)** SAM - 5J. **(c)** Detectron2 - 7.5J. **(d)** SAM - 7.5J.

**Figure 11:**
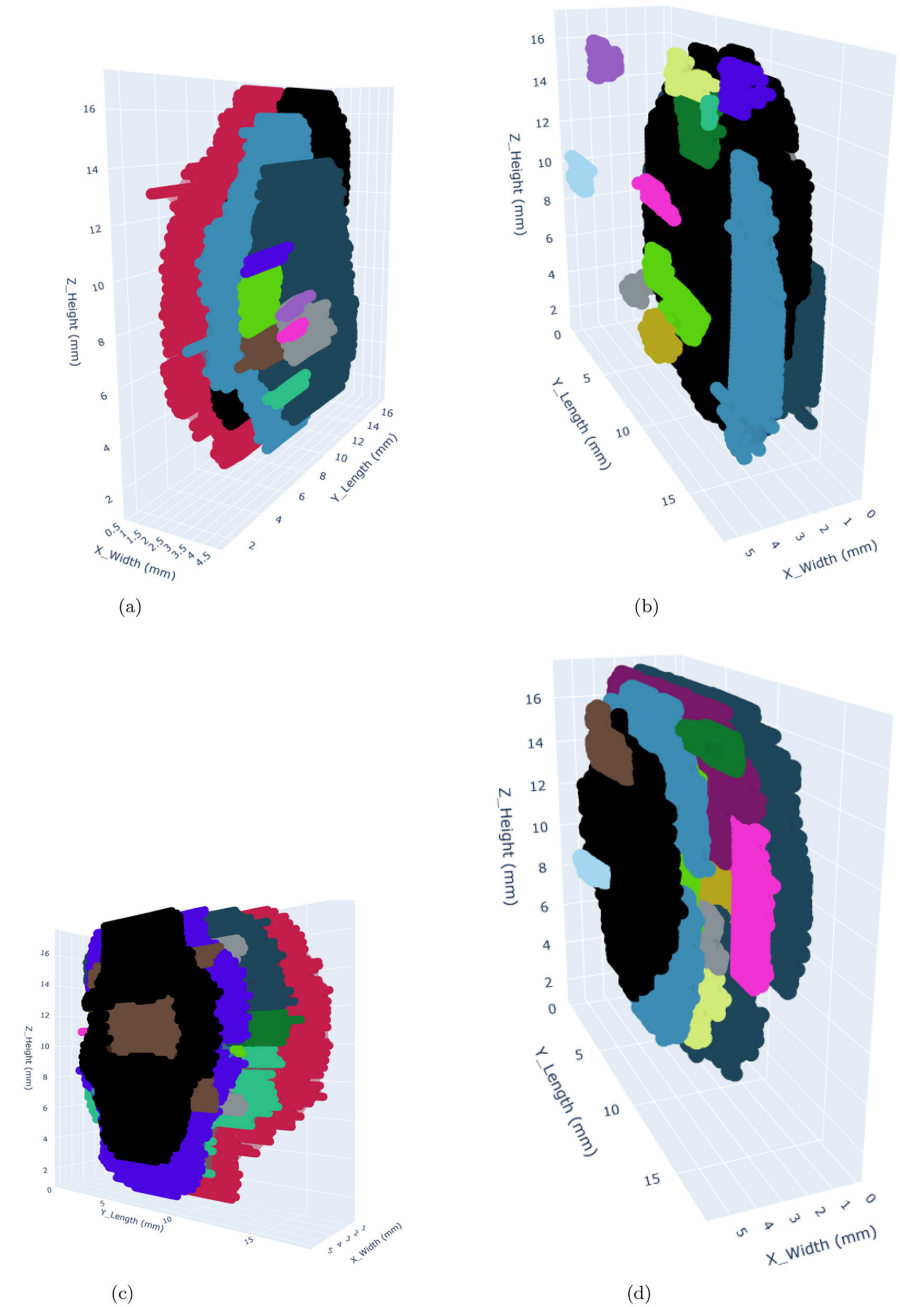
3D visualization of damages within the FML plates occuring at 10J and 12.5J impact energy levels using Detectron2 (left) and SAM (right). The clusters with differences in shape and size highlight the segmentation capabilities of both models. The colors used to represent the clusters are arbitrary and only used to distinguish different clusters from each other. **(a)** Detectron2 - 10J. **(b)** SAM - 10J. **(c)** Detectron2 - 12.5J. **(d)** SAM - 12.5J.

The segmented damage masks generated by both models were used to create a 3D damage point cloud by stacking the slices along the z-direction. The DBSCAN algorithm was then applied to form clusters of damage. DBSCAN (Density-Based Spatial Clustering of Applications with Noise) was chosen as the clustering algorithm for this study because of its suitability for analyzing irregularly shaped damage clusters in 3D point cloud data. In contrast to the other clustering algorithms, it does not require the number of clusters to be specified beforehand and is particularly effective in handling noise, which is critical given the presence of false positives in the segmented damage masks. It is advantageous to use it, particularly for this study, because it identifies and isolates outliers (false positives, especially in the case of SAM) as noise, which helps in minimizing their influence on the clustering process. This is crucial for distinguishing genuine damage clusters from spurious ones. It is able to identify the clusters of arbitrary shapes, which makes it well-suited for modeling the complex geometries of damage patterns in FML plates. Additionally, the clustering algorithm can be adapted to different energy levels by tuning the parameters such as (*eps* and *min_samples*). However, it has certain limitations as well. Its performance strongly depends on the choice of *eps* and *min_samples*. While *min_samples* was kept constant, varying *eps* values had to be determined experimentally for each energy level, adding complexity to the analysis. Its sensitivity to dense regions may have led to the exacerbated tendency in the case of SAM to produce numerous small clusters (many were false positives). It can become computationally expensive to process large datasets, particularly when generating a high number of clusters, as observed with SAM. The minimum samples (*min_samples*) value used for all energy levels is 2. At the 5J energy level, the *eps* value for SAM and Detectron2 is 0.286 μm and 0.344 μm, respectively. At the 7.5J and 10J energy levels, both SAM and Detectron2 have an *eps* value of 0.286 μm. At the 12.5J energy level, the *eps* value for SAM is 0.189 μm, and for Detectron2, it is 0.286 μm. The concave hull was fitted over the identified clusters, which were used for further analysis. The alpha value of 1.5 produced the best hull fitting results for all the impact energy levels. The clustered point clouds representing the damages are presented in the Figures 10, 11. A significant difference in the identified damage clusters is observed. SAM shows a tendency to produce a higher number of small clusters, many of which were identified as false positives. In contrast, in the case of the Detectron2, cleaner results that were more closely aligned with the expected damage cluster patterns were obtained. This discrepancy is particularly evident in the areas outside the plate boundaries, such as the cluster with black color observed in the 7.5J impact case for SAM lies completely outside the FML plate. In both cases, a positive correlation between impact energy and damage characteristics was observed. As the impact energy increased, a corresponding rise in both the size and number of damage clusters was observed, consistent with our expectations. Ideally, the maximum number of resulting clusters should approximate two damages per prepreg layer, totaling eight for our four prepreg layer configuration of plates. This pattern was observed during manual annotation of the CT slices, which were used for the training of the models. However, in both cases, SAM and Detectron2, there is a deviation from this ideal scenario. SAM generated a significantly higher number of clusters, ranging from 66 to 164 for each energy level, with over 75% of these clusters having a volume less than 0.1 mm as illustrated in Figure 14. This is most likely due to the false positives generated in large numbers as shown in Figures 7, 8. In contrast, the Detectron2 produced a more conservative number of clusters, ranging from 5 to 18, with the majority having volumes exceeding 0.1 mm^3^ across all energy levels. Detectron2 provides a cluster distribution more closely aligned with the expected damage patterns in FML plates. The number of clusters is still higher in Detectron2 than the expected number. It is likely due to the following reasons:

1. **Suboptimal hyperparameters:** in some instances, larger damage clusters were not properly identified by DBSCAN, possibly due to suboptimal hyperparameters. This resulted in the fragmentation of larger clusters into smaller components.
2. **Discontinuous predictions:** visual inspection revealed that some smaller clusters appeared to form parts of larger damage areas. This was likely due to missing damage predictions by the model in intermediate slices, creating an illusion of discontinuity. For example, in the 5J impact energy case using Detectron2, six distinct clusters were identified. Notably, around x = 4 (Figure 10), three smaller clusters were observed, potentially resulting from true positive predictions in specific slices. Further analysis of slice continuity suggested that these three clusters were likely part of a larger, continuous damage area within the FML plate.

**Figure 12:**
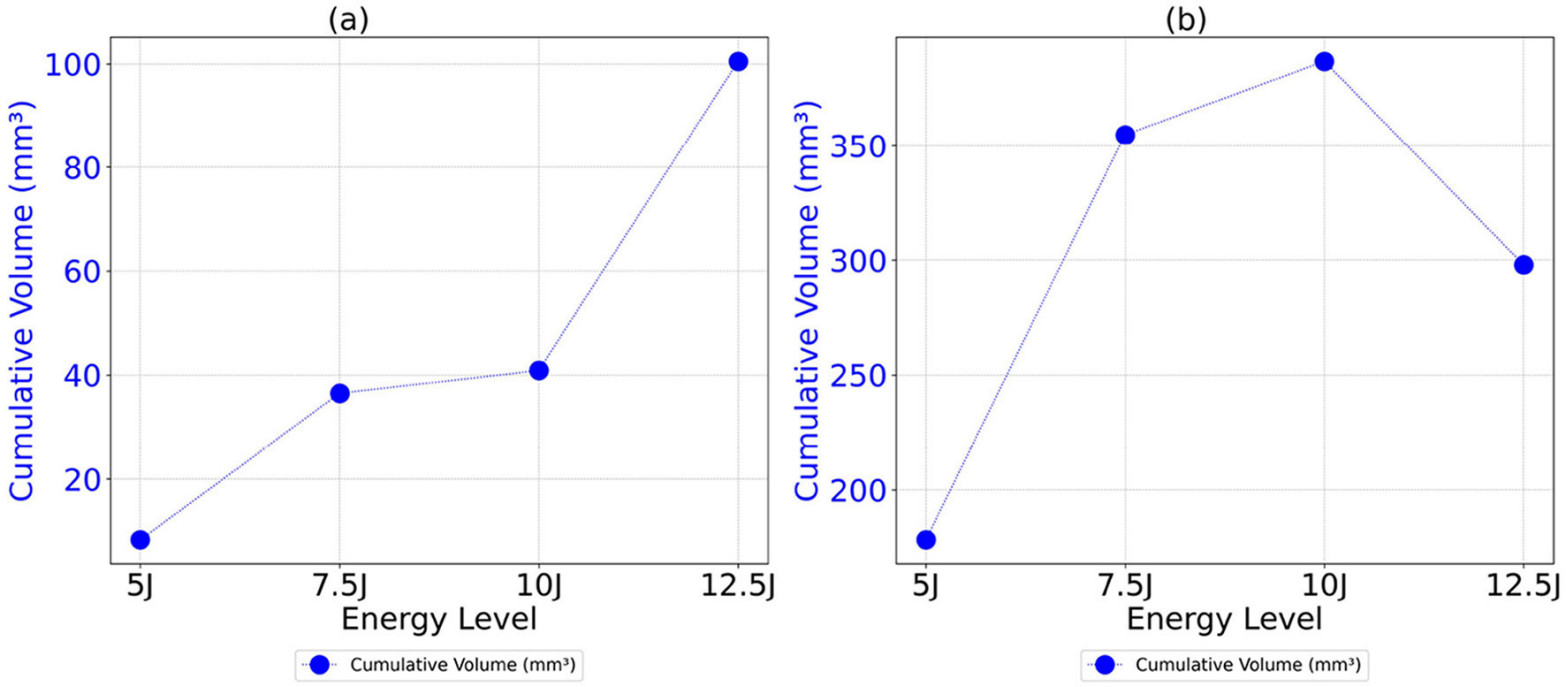
Cumulative volume as a function of energy level for both Detectron2 and SAM models. **(a)** Detectron2. **(b)** SAM.

**Figure 13:**
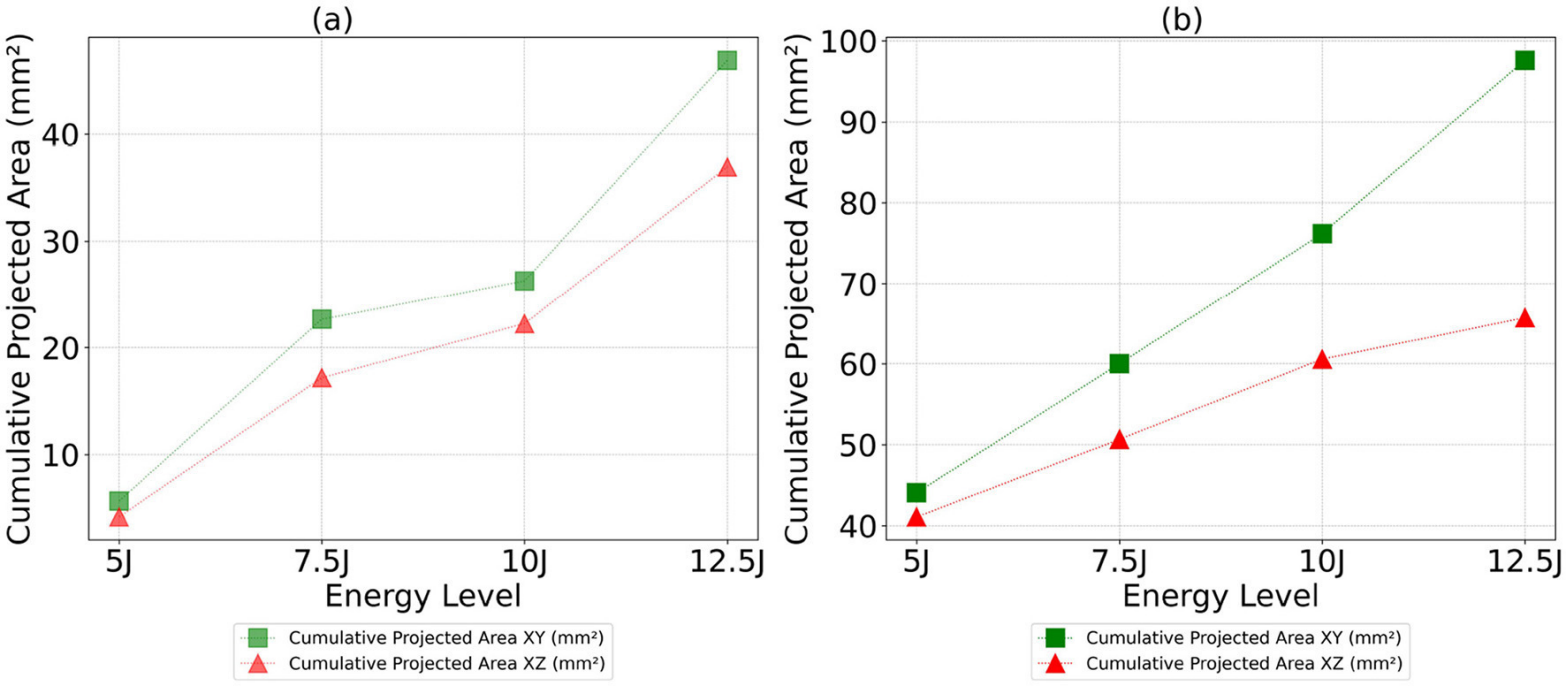
Cumulative projected areas in the XY and XZ planes as a function of energy level for both Detectron2 and SAM models. **(a)** Detectron2. **(b)** SAM.

The analysis of cumulative volume and the projected areas in the XY and XZ planes reveals a general trend of increasing damage sizes with higher impact energy levels for both Detectron2 and SAM, as shown in Figures 12, 13, aligning with expected outcomes. However, an anomaly is observed in the SAM results at the 12.5J energy level, where the cumulative volume unexpectedly decreases compared to the 7.5J and 10J levels, despite an increase in projected areas XY and XZ planes. This discrepancy, likely due to underestimation of damage thickness, raises concerns about the reliability of SAM's predictions. Additionally, SAM consistently produces larger cumulative volume and projected area values compared to Detectron2, which may be attributed to a higher number of false positives in SAM's predictions. These findings suggest that while both models show some correlation with impact energy, SAM's results should be interpreted with caution due to potential overestimation of volumes and the area calculations, and inconsistencies in damage assessment. Furthermore, SAM exhibits substantially higher standard deviations for cumulative volumes and area values when calculations are repeated five times, as shown in Tables 2, 3, 4, indicating lower reliability compared to Detectron2. Interestingly, Detectron2 shows a trend of increasing standard deviation as impact energy increases, suggesting a decrease in reliability for higher impact energy. However, this pattern is not observed in SAM's results, which show no noticeable trend in standard deviation across different energy levels for the volume as well as the area calculations. These observations further highlight the superior consistency of Detectron2 in damage assessment, particularly at lower impact energies, while highlighting the unpredictable nature of SAM's outputs across the entire range of impact energies tested.

**Table 2:** Standard deviation (obtained by the five different experimental runs) of the various statistical measures: cumulative volume, mean volume, maximum volume, etc. across different energy levels.

**Statistic (mm^3^)**	**Detectron2**				**SAM**			
	**5J**	**7.5J**	**10J**	**12.5J**	**5J**	**7.5J**	**10J**	**12.5J**
Cumulative	1.3900	1.3500	13.4100	42.0000	157.4000	295.8500	253.3300	214.6100
Mean	0.3200	1.1100	1.2800	2.1700	1.2200	3.6600	3.4800	1.6600
Min.	0.0200	1.1600	0.0100	0.0002	0.0000	0.0000	0.0000	0.0001
25th Per.	0.0500	1.3600	0.0400	0.0100	0.0010	0.0019	0.0012	0.0004
Median	0.1800	1.6100	0.3900	1.9200	0.0100	0.0040	0.0100	0.0019
75th Per.	0.8400	0.4800	0.8900	1.2100	0.0300	0.0100	0.0800	0.0100
Max.	0.6300	1.6200	15.3500	38.2400	103.9800	302.9700	245.9700	124.1800

**Figure 14:**
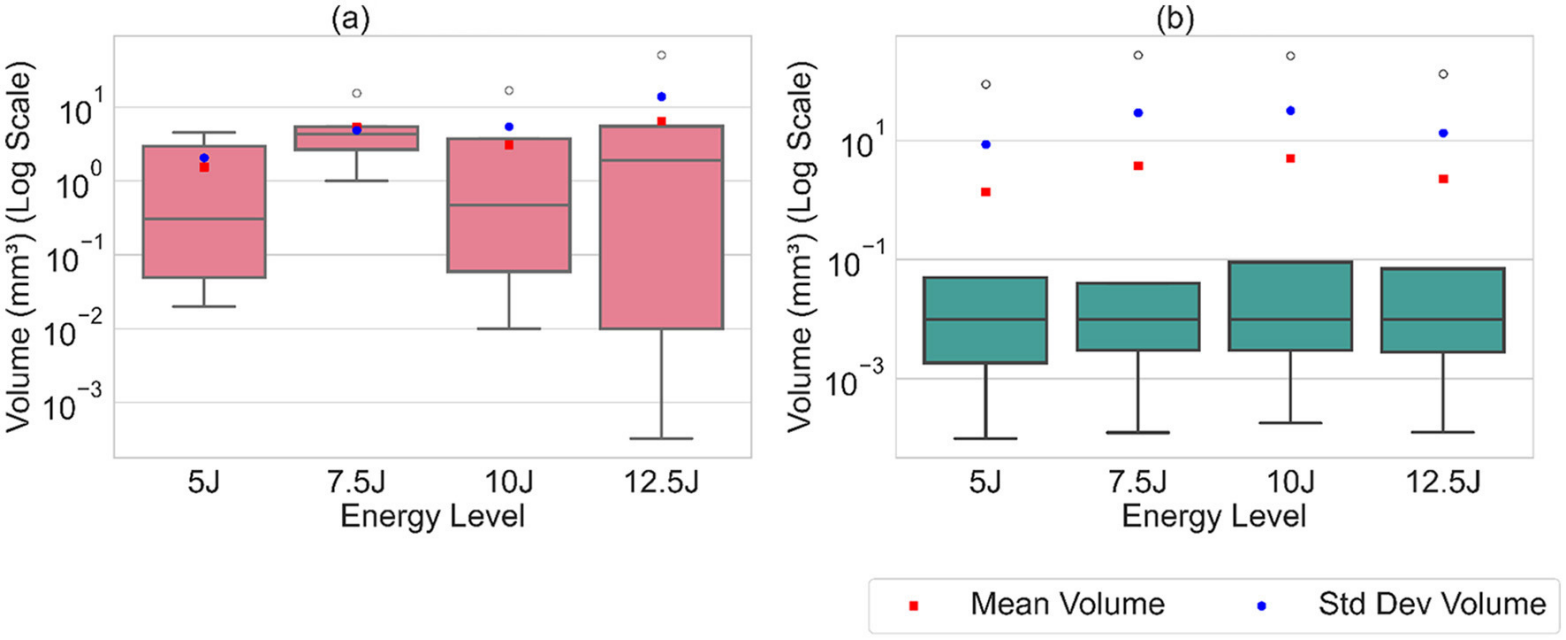
The box plot of calculated volume values along with the mean and standard deviation, illustrating the distribution of cluster volumes. **(a)** Detectron2. **(b)** SAM.

The corresponding calculated statistical measures are shown in Figure 14.

**Table 3:** Standard deviation (calculated from five experimental runs) of various statistical measures (projected areas) in the XY and XZ planes at different impact energy levels, using Detectron2.

**Statistic (mm^2^)**	**Detectron2** **XY Plane**				**Detectron2** **XZ Plane**			
	**5J**	**7.5J**	**10J**	**12.5J**	**5J**	**7.5J**	**10J**	**12.5J**
Cumulative	0.1200	0.6900	1.3700	3.9500	0.0700	0.4300	2.9100	3.1400
Mean	0.0800	0.6200	0.2600	0.2700	0.0600	0.5300	0.3300	0.1800
Min.	0.0400	1.0200	0.0500	0.0044	0.0300	0.9200	0.0200	0.0026
Median	0.0900	0.4900	0.2800	1.6600	0.0700	0.6400	0.4000	1.1700
25th Perc.	0.0400	1.0000	0.0800	0.0100	0.0500	0.9000	0.0200	0.0100
75th Perc.	0.1500	0.2700	0.4300	0.4500	0.1400	0.3100	0.6800	0.7500
Max.	0.0400	0.2500	2.8200	4.8800	0.0100	0.1600	2.8000	5.1400

**Figure 15:**
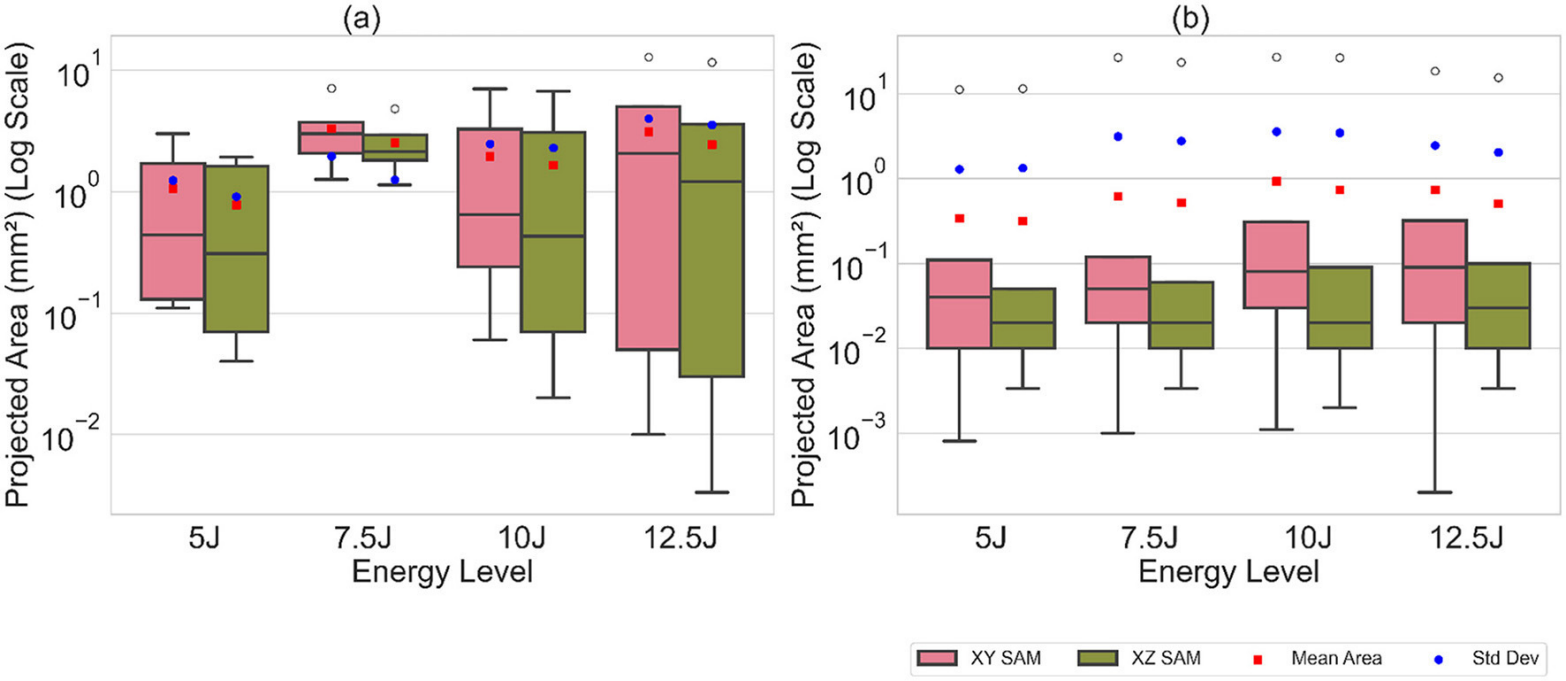
The box plot of the calculated projected areas in XY, XZ plane along with the mean and standard deviation. **(a)** Detectron2. **(b)** SAM.

The corresponding statistical measures are presented in Figure 15.

**Table 4:** Standard deviation (calculated from five experimental runs) of various statistical measures (projected areas) in the XY and XZ planes at different impact energy levels, using SAM.

**Statistic (mm^2^)**	**SAM** **XY Plane**				**SAM** **XZ Plane**			
	**5J**	**7.5J**	**10J**	**12.5J**	**5J**	**7.5J**	**10J**	**12.5J**
Cumulative	25.5500	19.5500	19.8100	29.6800	22.3000	16.9700	12.9500	13.4000
Mean	0.2000	0.2700	0.3700	0.1900	0.1800	0.2200	0.2800	0.1300
Min.	0.0004	0.0010	0.0007	0.0003	0.0000	0.0000	0.0016	0.0000
Median	0.0200	0.0200	0.0400	0.0200	0.0028	0.0020	0.0100	0.0041
25th Per.	0.0100	0.0100	0.0100	0.0039	0.0013	0.0019	0.0018	0.0022
75th Per.	0.0500	0.0300	0.1700	0.0500	0.0200	0.0100	0.0600	0.0200
Max.	9.3300	22.7800	18.5200	10.3100	8.6400	19.7100	16.1600	8.5000

The corresponding statistical measures are presented in Figure 15.

The distribution of volume values and projected areas (minimum, 25th percentile, median, 75th percentile, maximum, mean) for each energy level is illustrated in Figures 14, 15, highlighting distinct patterns between Detectron2 and SAM results. Detectron2 demonstrates a wider spread of volume and area values. The presence of low minimum values for the volumes and projected areas indicates the detection of some very small clusters, possibly due to cluster continuity issues discussed earlier. Generally, the spread increases with impact energy for Detectron2, except at 7.5J, where a notably smaller distribution suggests more uniform cluster sizes and fewer fragmented clusters. In contrast, SAM shows a narrower spread of values concentrated at the lower end, coupled with higher maximum values. This pattern indicates that SAM detects numerous smaller clusters with low volumes and projected areas, alongside a few disproportionately large clusters. The mean volume and projected areas increase with impact energy for both models, confirming the expected trend of larger damages at higher energies. The Tables 2, 3, 4 present the standard deviations of calculated values over five runs. SAM generally shows smaller standard deviations for minimum and quartile values, likely due to consistent detection of smaller clusters across runs. However, SAM exhibits larger standard deviations for mean and maximum values compared to Detectron2, indicating less consistency in detecting larger clusters. These findings suggest that while SAM may be more consistent in detecting smaller damages, Detectron2 offers a more balanced and reliable detection across various cluster sizes, particularly for larger damaged areas.

**Figure 16:**
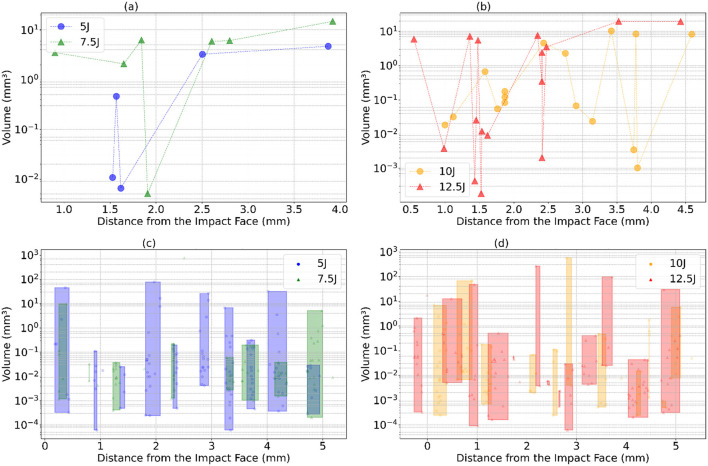
Damage cluster volumes obtained for the increasing mean distance from the impact face of the plate for different impact energies. **(a, b)** shows the damage cluster for the detectron2. **(c, d)** shows a large number of clusters in the case of SAM. They are grouped inside the rectangles for better representation. **(a)** Detectron2: 5J and 7.5J. **(b)** Detectron2: 10J and 12.5J. **(c)** SAM: 5J and 7.5J. **(d)** SAM: 10J and 12.5J.

The damage cluster volumes and their average distance from the impact face for one of the runs are illustrated in Figure 16. It was observed that SAM detects a larger number of clusters compared to Detectron2. Generally, damage volumes increase with the increasing distance from the impact face, although some dips occur, likely due to cluster fragmentation issues previously discussed. At the 10J and 12.5J energy levels, there is a greater range of fluctuations in damage volumes than at lower energy levels, suggesting increased cluster fragmentation with higher impact energy. Notably, the first damage cluster appears at around 1.5 mm for the 5J impact energy and decreases to 0.5 mm for the 12.5J impact energy. Similarly, the last damage cluster is observed around 3.9 mm for the 5J and at around 4.5 mm for the maximum impact energy, indicating more widespread damage within the material as energy increases. For both 5J and 7.5J, SAM shows significant variability in damage volumes across the range of distances, unlike Detectron2, which has more pronounced spikes. SAM's plots display frequent, smaller fluctuations in damage volume, suggesting numerous false positives or cluster fragmentations. Similar patterns are observed at 10J and 12.5J, with many small fluctuations in damage volume. The variance in damage volume is higher at higher energy levels, indicating more significant damage clusters or increased damage spread across different distances.

**Figure 17:**
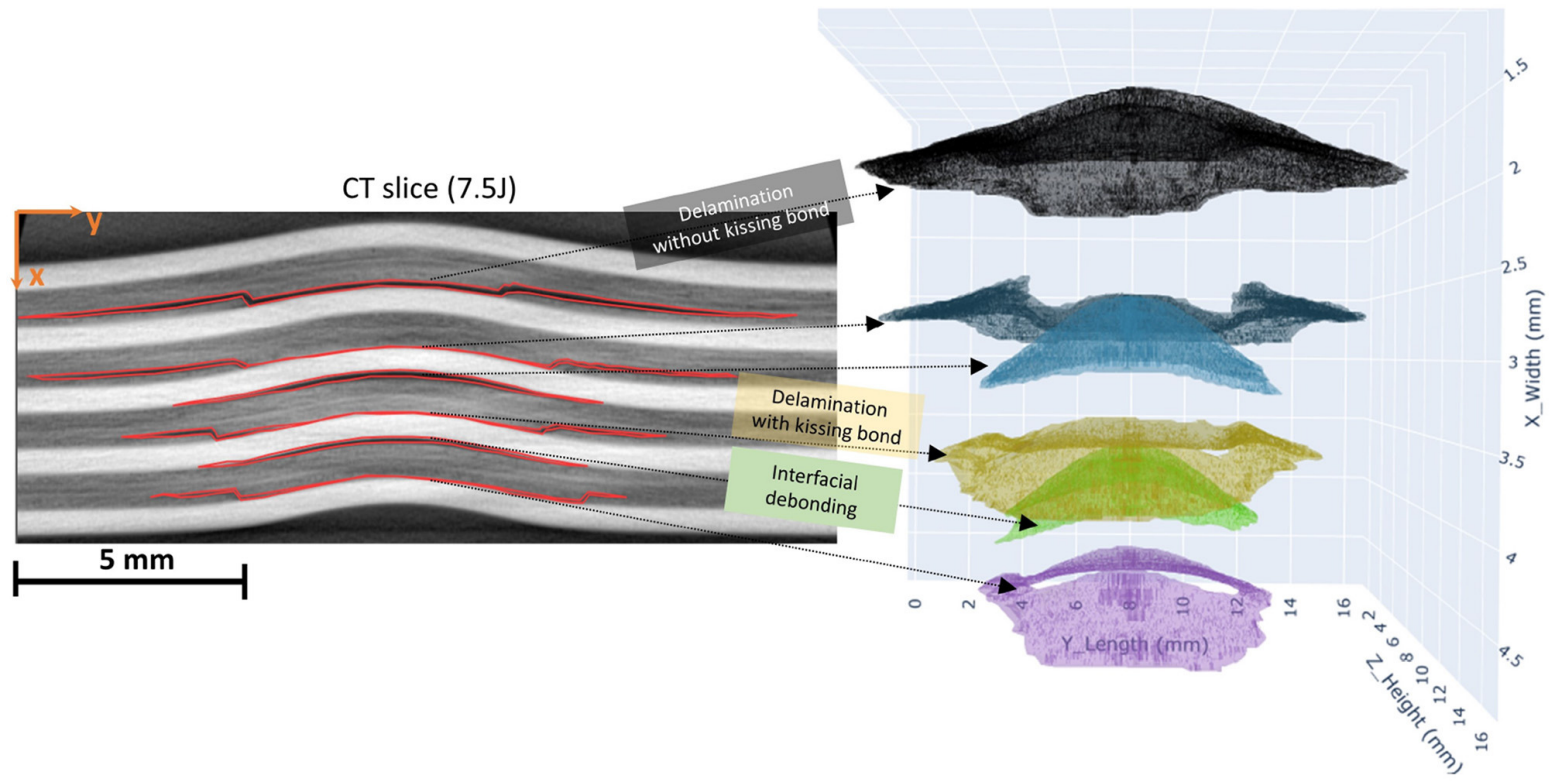
On the left side the damages annotated on the slices (2D) are shown which are stacked and clustered to form damages in 3D shown on the right. Three types of damage cluster shapes were identified: delaminations with kissing bond (violet, yellow, and deep blue clusters), delaminations without kissing bond (black cluster), interfacial debonding (green, light blue).

It can also be observed that the damage accumulation increases on the non-impacted side of the FML plate as the impact energy increases. The segmented regions typically highlight the regions with interfacial debonding at the interfaces between the metal and the prepreg (fiber) layers and can be referred to as the main energy absorption mechanism during an impact. It has been found that the height of these interfacially debonded regions increases with the increase in impact energy, with the maximum extent on the non-impacted side of the laminate. In Figure 17, an example slice from the middle of the damaged plate with 7.5J impact energy is illustrated. Three distinct damage morphologies in the FML structure were observed. Interfacial debonding, represented by blue and green clusters, appears as smaller, bell-shaped formations. Delaminations with kissing bonds, shown in violet, yellow, deep blue, and black, are characterized by a central hole surrounded volume formed by delaminated areas between prepreg laminates. The hole, while not directly visible, indicates the presence of a kissing bond defect. Delaminations without kissing bonds are usually broader, complete bell-shaped, and typically larger than the interfacial debonding present in the same prepreg layer. These larger delaminations lack the central hole seen in their kissing bond counterparts. The retained plastic deformation in the metal layers increases with increasing impact energies. The metal layers behave elastically, requiring a certain threshold of impact energy to enter the plastic deformation regime. If the impact energy is below this threshold, there won't be any signs of induced damage, and the damage mechanisms are limited to microcracking within the fiber layers (prepregs). This cracking includes matrix cracking and debonding at the fiber and matrix interfaces. As the impact energy increases, the metal layers inhibit plastic deformation while the prepreg layers still hold to the elastic regime. These prepreg layers or the fiber layers have debonded from the metal layer as a result of the impact and are now resting over the deformed metal layers, leading to kissing bonds. These rings highlight the close contact between the metal and the prepreg layers at the center, giving it the shape of a circular ring. These kissing bonds have no substantial adhesive bonding between the prepreg and the metal layers. The plastic deformation within the metal layers increases with the increasing impact energy, which is consistent with the damage segmentation results that show greater damage accumulation with higher impact energy. These segmented regions, as mentioned earlier, correspond to the regions with interfacial debondings between the metal and the fiber layers. These are induced via shear-induced matrix cracks resulting from the shear loads within the prepreg layers. These cracks upon reaching the interface at the metal lead to interfacial debondings, while on the other end of it, these lead to internal delaminations within the fiber layers. These delaminations always take place in between the cross-plies, and the regions with interfacial debondings increase with an increase in impact energy as the damage spreads across the laminate. This is evident from the findings, as the segmented damage regions increase with the impact energy. The energy absorption mechanism at higher impact energies shifts to the retained plastic deformation in the metal layers. The maximum extent of the interfacial debonding takes place at the non-impacted side of the laminate, specifically in between the outermost metal layer on the non-impacted side and the underlying prepreg. This debonding occurs after the maximum deflection of the FML specimen during impact. At this point, the kinetic energy of the impactor has been dissipated within the FML, and where the stored elastic strain energy starts to transfer back into the laminate and the impactor. The elastic prepreg layers release this stored energy until the point it returns to the full straightening or until the fiber breakage takes place. This difference in the behavior of the metal and prepreg layers during the rebound stage in an event of an impact leads to peeling at the interface, thus resulting in interfacial debonding (Pärnänen et al., 2015). In previous studies, it has been reported that this metal and prepreg layer debonding is the main damage mode, especially in the case of laminates with lower metal volume fraction where the damage mode is predominantly governed by the elastic fiber layers (Nakatani et al., 2011; Fan et al., 2011). It is also to be noted that by increasing the number of interfaces within the laminate, the extent of these interfacial debondings can be manipulated. The bending stiffness of the prepreg layers can influence the extent of these debonded regions, arguably with higher stiffness leading to higher peeling forces at the interfaces. These debondings are a result of the different tendencies of the constituting elastic-plastic metal and elastic prepreg layers relating to out-of-plane deformation during an impact, leading to peeling forces at the interfaces, thus, to interfacial debonding (Pärnänen et al., 2015).

### 4.1 Synthetic data

Obtaining the exact ground truth for damage characteristics remains a challenge, limiting our ability to accurately quantify the errors introduced during the calculation process. To address this challenge, a simulated impact-damaged plate and the corresponding simulated CT slices were generated to create synthetic ground truth (GT) data as explained in the Section 2.3. This GT data serves as a reference to evaluate the errors in the calculated volumes of the damaged regions. For damage segmentation, Detectron2 was used as it performs better than SAM in all aspects, as observed in the analysis above. The deep learning model was trained using a dataset of 11 images, each with a resolution of 788x237 pixels, and validated using 4 images of the same resolution. The early stopping technique was applied to prevent overfitting. The model's performance was evaluated using the same performance metrics (IoU, Precision, Recall, F1 score) used to compare the Detectron2 and SAM. The results are summarized in the Table 5.

**Table 5:** Performance metric for the Detectron2 model trained using the synthetic data.

**Performance metric**	**Average score (% ± Std. Dev.)**
IoU	64.80 ± 0.52
Precision	93.53 ± 0.26
Recall	68.38 ± 0.49
F1 Score	79.01 ± 0.35

**Figure 18:**
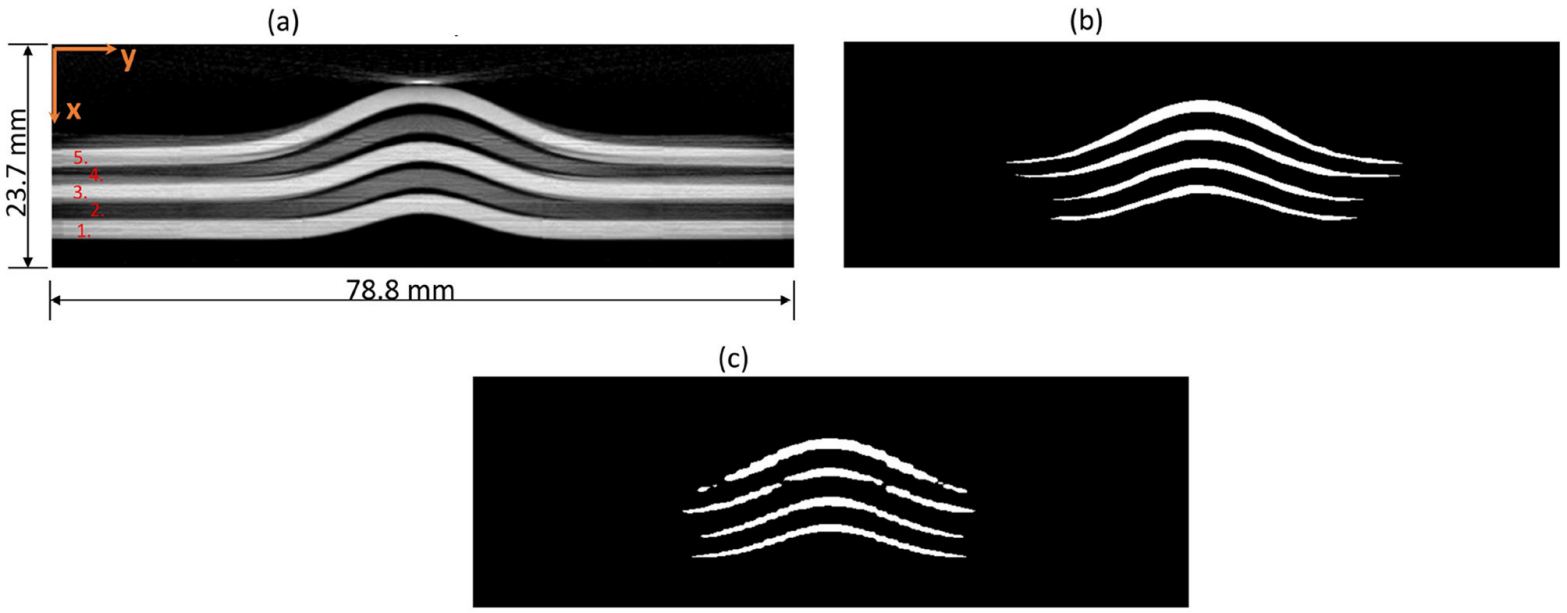
**(a)** An example CT slice of synthetic data along with the layer numbering shown in red text. **(b)** The hand-annotated mask. **(c)** The mask predicted by the detectron2.

Figure 18 presents an example slice of the synthetic CT data along with the corresponding mask. The segmented masks were stacked to generate a point cloud representing the damaged regions. Subsequently, the DBSCAN algorithm (*eps* = 11, *min_samples* = 2) was employed to cluster the segmented damage regions, followed by fitting a concave hull using alpha shape (*alpha* = 1.5) to estimate the volume of each cluster. The summary of the volume results is provided in the Table 6.

**Table 6:** Comparison of the calculated delamination volume using the process pipeline with the Detectron2 framework and ground truth delamination volumes.

**Delamination**	**Detectron2 +**	**Ground**	**Error**	**Percentage**
**Region**	**DBSCAN (mm^3^)**	**Truth (mm^3^)**	**(mm^3^)**	**Error (%)**
Layer (1,2)	283.87 (± 10.17)	221.12	62.75	28.38%
Layer (2,3)	338.18 (± 9.54)	381.68	43.50	11.40%
Layer (3,4)	490.05 (± 12.47)	644.32	154.27	23.94%
Layer (4,5)	659.25 (± 15.70)	1055.18	395.93	37.52%
Total	1771.35	2302.30	656.45	**28.51%**

The error of 28.51% was observed using the synthetic data as shown in Table 6. The potential sources of error in the volume calculations arise from several factors. First, the hand annotations used for training and validating the model may contain inaccuracies, introducing initial bias. Second, the model's prediction scores, being < 1, indicate that the model does not fully capture all damaged regions, leading to incomplete segmentation. Finally, the process of fitting the predicted damage point clouds with a concave hull to generate 3D meshes for volume estimation introduces additional errors, as this method may not perfectly represent the actual geometry of the delamination regions.

### 4.2 Integrated gradients

**Figure 19:**
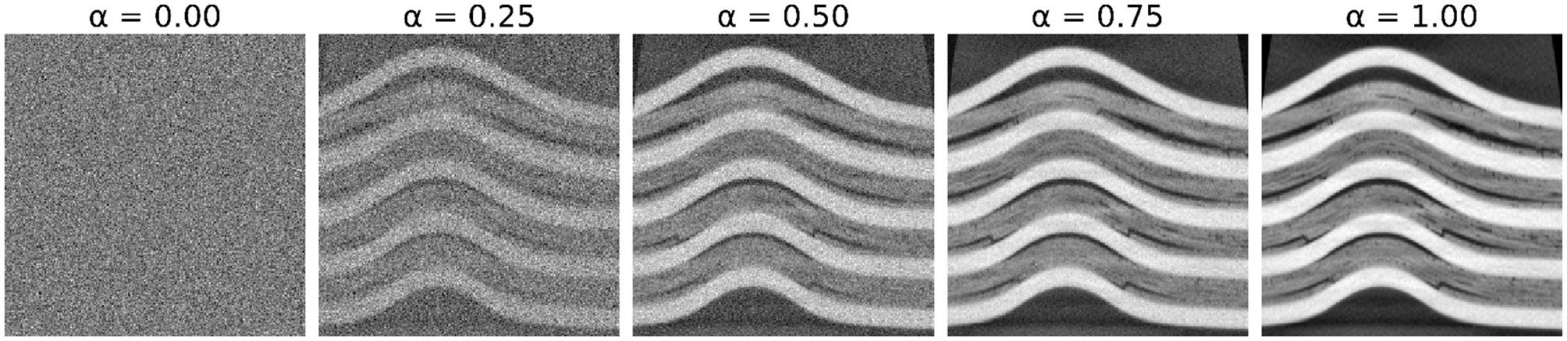
The interpolated images are generated by increasing the alpha which are used as an input. The attribution scores at each step are calculated and added up to get the final attribution map.

The Gaussian noise is selected as a baseline (presented in Figure 19), which has near-zero attribution scores when tested with Detectron2 and SAM. The experiment is conducted with 20 steps.

**Figure 20:**
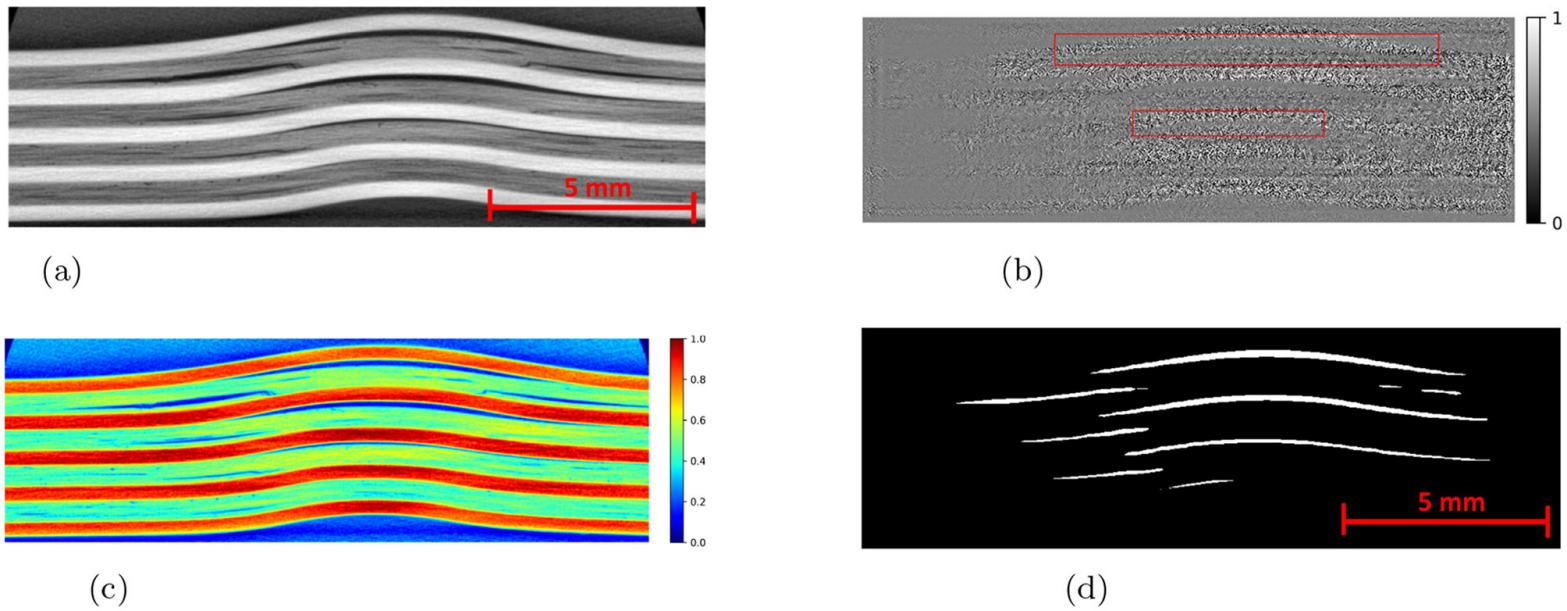
Visualization of IG results for impact energy of 10 Joules. **(c)** Shows the attribution map produced using the IG method with the modified loss function applied on the trained Mask R-CNN model, using the CT slice of the FML plate shown in **(a)**. Example red boxes shown in attribution map **(b)** highlight attribution of damages. **(a)** CT slice with impact energy of 10 Joules. **(b)** Attribution map overlay. **(c)** Attribution map (modified loss). **(d)** Predicted mask.

**Figure 21:**
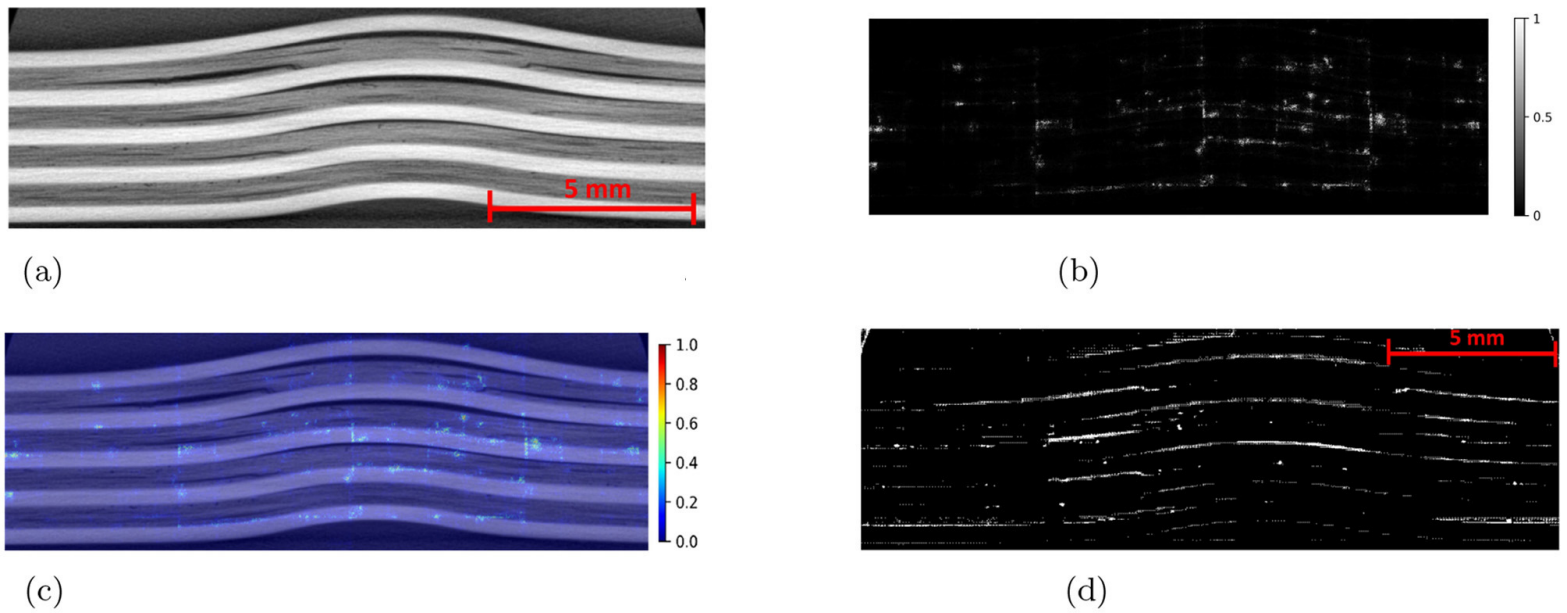
An example CT slice of the damaged plate with 10 Joules impact energy is shown in **(a)**. The attribution map **(b)** is produced by applying the IG method to the trained SAM. The overlap of the attribution map and CT slice in **(c)** suggests that the SAM has very low interpretability. **(a)** CT slice with impact energy of 10 Joules. **(b)** Attribution map. **(c)** Overlap of attribution map and CT slice. **(d)** Predicted mask.

**Figure 22:**
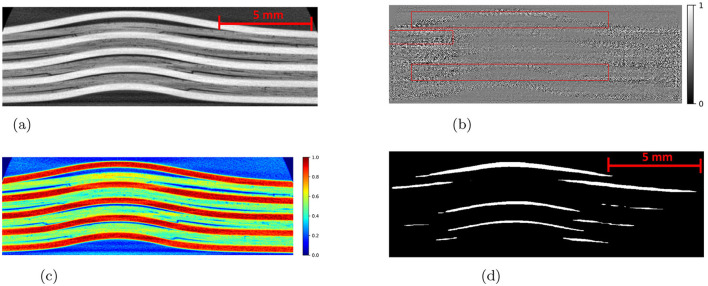
Visualization of IG results for impact energy of 12.5 Joules. **(c)** Shows the attribution map produced using the IG method with the modified loss function applied on the trained Mask R-CNN model, using the CT slice of the FML plate shown in **(a)**. Example red boxes shown in attribution map **(b)** highlight attribution of damages. **(a)** CT slice with impact energy of 12.5 Joules. **(b)** Attribution map overlay. **(c)** Attribution map (modified loss). **(d)** Predicted mask.

**Figure 23:**
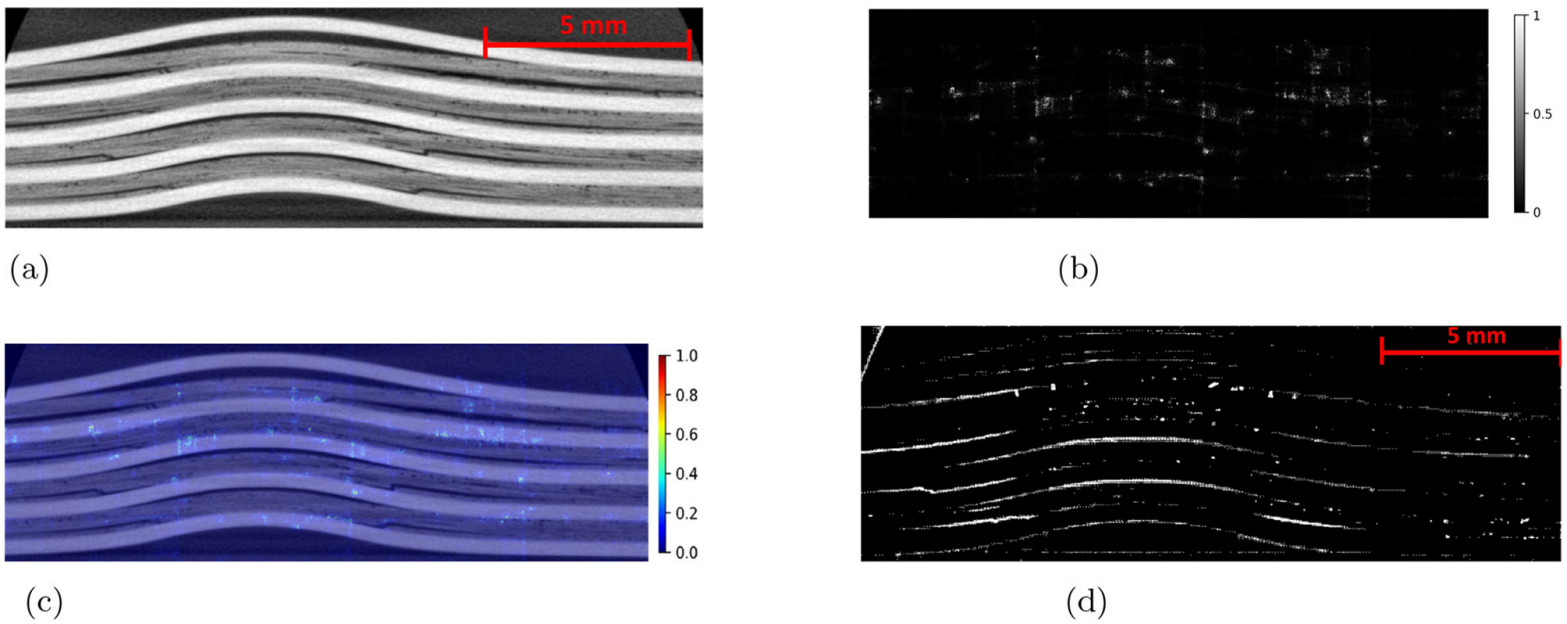
CT slice of the damaged plate with 12.5 Joules impact energy is shown in **(a)**. The attribution map is produced by applying the IG method to the trained SAM. The overlap of the attribution map and CT slice in **(c)** suggests that the SAM has very low interpretability. **(a)** CT slice with impact energy of 12.5 Joules. **(b)** Attribution map. **(c)** Overlap of attribution map and CT slice. **(d)** Predicted mask.

**Figure 24:**
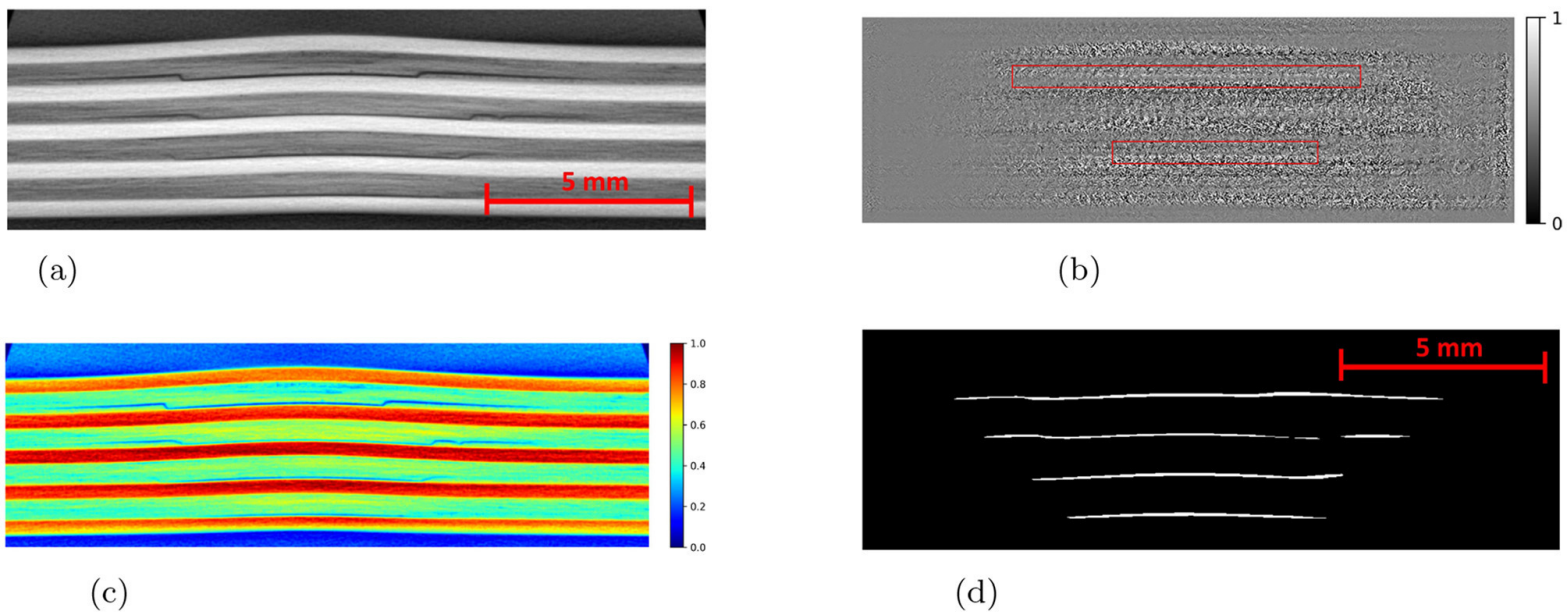
Visualization of results for impact energy of 7.5 Joules. **(c)** Shows the attribution map produced using the IG method applied on the trained Detectron2 model, using the CT slice of the FML plate shown in **(a)**. The model's predicted mask is displayed in **(d)**, highlighting its interpretability. Example red boxes shown in attribution map **(b)** highlight attribution of damages. **(a)** CT slice with impact energy 7.5 Joules. **(b)** Attribution map. **(c)** Attribution map (modified loss). **(d)** Predicted mask by Detectron2.

**Figure 25:**
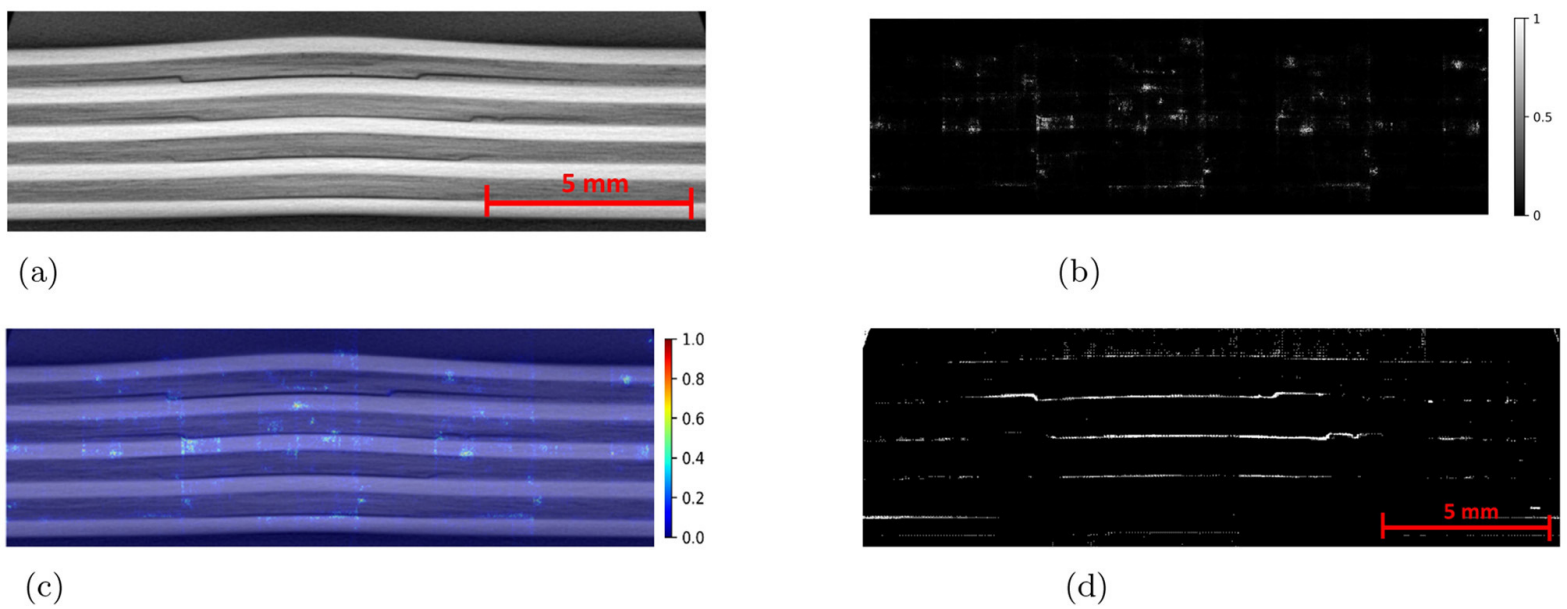
An example CT slice of the damaged plate with 7.5 Joules impact energy is shown in **(a)**. The attribution map is produced by applying the IG method to the trained SAM. The overlap of the attribution map and CT slice in **(c)** suggests that the SAM has very low interpretability. **(a)** CT slice with impact energy of 7.5 Joules. **(b)** Attribution map. **(c)** Overlap of attribution map and CT slice. **(d)** Predicted mask.

The CT slices from the energy levels 12.5 J, 10 J, and 7.5 J were selected randomly for the interpretability analysis. The results are presented in Figures 20–25. The attribution map results for Detectron2 are shown in Figures 20b, 22b,24b. When compared with the attribution map by SAM shown in Figures 21, 23, 25, it can be noted that the attribution maps of the detectron2 highlight the damage areas better. In the case of Detectron2, the high attribution regions are observed to be closer to the damaged areas than the non-damaged areas of the plate. The high attribution region, along with some noise, tends to surround the damaged features in the plate, and some separation between damaged and non-damaged regions can be observed. The damages are relatively free from the attribution noise, highlighting the focus of the Mask R-CNN model in identifying the damages. The attribution maps in both SAM and Detectron2 are able to identify the plate region from the outside region. When comparing the attribution map of both the SAM and Mask R-CNN, the sensitivity to noise is lower in the Mask R-CNN. The attribution results shown in Figures 21, 23, 25 highlight a few important areas having high attribution values, sparsely distributed, which are mostly concentrated on the metal layers. This suggests that SAM might be using the metal layer as a reference. There are no coherent patterns that can be identified, so it is hard to identify which input features are influencing the model's decision. The SAM has limited transparency compared to Detecron2, which is a significant disadvantage when it is used for damage detection within FML. Overall, the gradient maps in both models show that there is no complete explainable correlation between input images and output masks. The attribution maps shown in Figures 20c, 22c,24c were produced using a modified loss function used during the inference from the Mask R-CNN model. The modified loss function sums up the linear input pixel intensities. The attribution maps with conspicuous color coding that highlights the metal layers, with the red and orange colors indicating high attribution values of more than 0.75. The interfaces between the metal layers and the prepreg layers can be identified with the yellow-colored regions. The prepreg layers are represented with a mix of green and yellow colored regions. The damages with the low attribution values are represented by the color blue. The regions outside the plate also have low attribution scores, thus represented by the deep blue shades. The attribution maps show the enhanced damaged regions clearly distinguishable from the metal plate regions. However, this method also marks the other artifacts present in the prepreg layers with similar attribution values as the damages, making the decision-making unclear. This makes it unreliable for defect detection explainability. The damage prediction masks by Detectron2 are shown in Figures 20d, 22d,24d, whereas for the SAM, the damage masks are shown in Figures 21, 23, 25, respectively.

## 5 Conclusion

The paper proposed a method to automate the damage detection, segmentation, and characterization in Fiber Metal Laminate (FML) plates using the state-of-the-art DL models: SAM and Mask R-CNN (implemented using the Detectron2 framework). The impact damage itself is composed of different mechanical damages like delaminations and cracks on different layers of the FML plate. The strength of these sub-damage classes varies by impact energy and layer. The study compared the explainability of both models qualitatively. A comprehensive comparison between both models was done based on key performance metrics: IoU, Precision, Recall, and F1-score. Further, they were compared based on the training speed and inference speed. The experiment results suggest that Mask R-CNN has superior performance and is more reliable as compared to SAM. A novel noise filtering algorithm was introduced and applied to the SAM, which helped in improving the IoU score by around 30%, Precision by around 25%, Recall by around 37% and F1 score by around 25% but still, it falls short of the performance scores of Mask R-CNN. The Mask R-CNN achieved an IoU and Precision scores around 2 times better, Recall 1.16 times better, and F1 score 1.79 times better than SAM with the noise filtering algorithm applied. Moreover, Mask R-CNN achieved remarkable training speed, which was around 8 times faster, and inference speed approximately 80 times faster than SAM. In terms of quality of damage predictions, Mask R-CNN produced cleaner masks with minimal false positives, unlike SAM, which generated masks with higher false positives as well as false negatives. Furthermore, the damage characterization experiment revealed that both the number and size of damage clusters increase with higher impact energy levels, confirming that higher energy impacts cause more extensive damage. The explainability experiment produced relatively better interpretable attribution maps in the case of Mask R-CNN than SAM. These findings suggest that Mask R-CNN should be the model of choice in damage diagnostics applications in Fiber Metal Laminates and also hint that it might be a better image segmentation model in general. The lack of absolute ground truth data was the biggest limitation of this study, due to which the absolute quantitative comparison was not possible. An attempt was made to overcome this limitation by performing experiments on a synthetic dataset.

In this work, macro-scale defects and damages are detected automatically using highly complex and deep non-linear Machine Learning models. As an outlook, we plan to investigate the automated feature marking and characterization of micro-scale defects and damages in CT images, e.g., cracks. Using reverse gradient-based input feature marking showed the fundamental issue with explainable AI of the two models compared in this work. Even using a mathematical method to get an understanding, which parts of the input images that contribute to the output feature marking done by the Mask R-CNN and SAM models do not provide enough insights into what the models are predicting in detail. The gradient maps mostly show that there is no complete explainable correlation between input images and output masks, which would exist if the images were marked by a human expert. The gradient maps showed noisy activations, which could prevent micro-scale marking. The detailed insights into the damage diagnostics provided in this study are crucial for structural health monitoring and integrity assessments, thus enabling better prediction and prevention of structural failures in structures involving FML plates. Future research could compare the performance of the latest machine learning model with the Mask R-CNN using the performance metrics introduced in this paper. The explainability study could be further extended to include diverse methods that might give a better understanding of the decision-making of SAM. Moreover, Mask R-CNN could be used to explore the possibility of integration into real-time damage diagnostics systems. The development of more advanced algorithms for noise filtering and damage characterization will also be valuable for advancing the field of damage diagnostics.

## Data Availability

The raw data supporting the conclusions of this article will be made available by the authors, without undue reservation.
