# Supplementary material for: Investigation of deep learning approaches for automated damage diagnostics in fiber metal laminates using Detectron2 and SAM
Source: Front Artif Intell. 2025 Aug 25;8:1599345. doi: 10.3389/frai.2025.1599345 (PMC12417882; doi:10.3389/frai.2025.1599345)
Supplement: Supplementary file 1 [file Supplementary_file_1.pdf]

A Appendix

Algorithm 1: Pseudocode of the layer generation function `L()` and a rough sketch of the mass-spring simulation function `Simulate()`.

```
bodies = []
springs = []

function L(offsetY, D, S) {
  offsetNodes = bodies.length
  for (y = 0; y < H; y++) {
    for (x = 0; x < W; x++) {
      bodies.push({ x: x * l - X0, y: offsetY + y * l - Y0 })
    }
  }
  offsetFixedBottom = bodies.length

  for (x = 0; x < W; x++) {
    y = l * S * G(x, W / 2, D)
    bodies.push({ x: x * l - X0, y: offsetY + y - Y0 })
  }

  offsetFixedLeft = bodies.length

  for (y = 0; y < H; y++) {
    bodies.push({ x: 0 - X0, y: offsetY + y * l - Y0, fixed: true })
  }

  offsetFixedRight = bodies.length

  for (y = 0; y < H; y++) {
    bodies.push({ x: (W - 1) * l - X0, y: offsetY + y * l - Y0, fixed: true })
  }

  for (x = 0; x < W; x++) {
    springs.push({ b1: bodies[offsetNodes + x], b2: bodies[offsetFixedBottom + x],
                   len: 0, k: K1, hide: hideSpecialElements })
  }
  for (y = 0; y < H; y++) {
    springs.push({ b1: bodies[offsetNodes + y * W], b2: bodies[offsetFixedLeft + y],
                   len: 0, k: K1, hide: hideSpecialElements })
  }
  for (y = 0; y < H; y++) {
    springs.push({ b1: bodies[offsetNodes + (W - 1) + y * W], b2: bodies[offsetFixedRight + y],
                   len: 0, k: K1, hide: hideSpecialElements })
  }

  for (y = 0; y < H; y++) {
    for (x = 0; x < W; x++) {
      if (x < (W - 1)) {
        // East
        i = x + y * W,
        j = (x + 1) + y * W
        springs.push({ b1: bodies[offsetNodes + i], b2: bodies[offsetNodes + j],
                       len: l, k: K2, drawLine: drawLineSprings })
      }
      if (y < (H - 1)) {
        // North
        i = x + y * W,
        j = x + (y + 1) * W
```

```
        springs.push({ b1: bodies[offsetNodes + i], b2: bodies[offsetNodes + j],
                      len: l, k: K2, drawLine: drawLineSprings })
      }
      if (x < (W - 1) && y < (H - 1)) {
        // North-East
        i = x + y * W,
        j = (x + 1) + (y + 1) * W
        springs.push({ b1: bodies[offsetNodes + i], b2: bodies[offsetNodes + j],
                      len: d, k: K2, drawLine: drawLineSprings })
      }
      if (x < (W - 1) && y > 0) {
        // South-East
        i = x + y * W,
        j = (x + 1) + (y - 1) * W
        springs.push({ b1: bodies[offsetNodes + i], b2: bodies[offsetNodes + j],
                      len: d, k: K2, drawLine: drawLineSprings })
      }
    }
  }
}

// some example generations (three-layer plate)
L(0, D, S)
L(1 * l, D * Tx, S * Ty)
L(2 * l, D * Tx * Tx, S * Ty * Ty)

function Simulate() {
  Set all bodies net force to 0

  for (sp of springs) {
    for (b of connectedTo(bodies, sp)) {
        calculate the force of the spring
        add the force to the body
    }
  }
}
```
